# Establishment of an Inactivation Method for Ebola Virus and SARS-CoV-2 Suitable for Downstream Sequencing of Low Cell Numbers

**DOI:** 10.3390/pathogens12020342

**Published:** 2023-02-17

**Authors:** Judith Olejnik, Juliette Leon, Daniel Michelson, Kaitavjeet Chowdhary, Silvia Galvan-Pena, Christophe Benoist, Elke Mühlberger, Adam J. Hume

**Affiliations:** 1 Department of Microbiology, Boston University School of Medicine, Boston, MA 02118, USA; 2 National Emerging Infectious Diseases Laboratories, Boston University, Boston, MA 02218, USA; 3 Department of Immunology, Blavatnik Institute, Harvard Medical School, Boston, MA 02115, USA; 4 INSERM UMR 1163, Institut Imagine, University of Paris, 75015 Paris, France; 5 Center for Emerging Infectious Diseases Policy & Research, Boston University, Boston, MA 02118, USA

**Keywords:** Ebola virus, SARS-CoV-2, virus inactivation, sequencing

## Abstract

Technologies that facilitate the bulk sequencing of small numbers of cells as well as single-cell RNA sequencing (scRNA-seq) have aided greatly in the study of viruses as these analyses can be used to differentiate responses from infected versus bystander cells in complex systems, including in organoid or animal studies. While protocols for these analyses are typically developed with biosafety level 2 (BSL-2) considerations in mind, such analyses are equally useful for the study of viruses that require higher biosafety containment levels. Many of these workstreams, however, are not directly compatible with the more stringent biosafety regulations of BSL-3 and BSL-4 laboratories ensuring virus inactivation and must therefore be modified. Here we show that TCL buffer (Qiagen), which was developed for bulk sequencing of small numbers of cells and also facilitates scRNA-seq, inactivates both Ebola virus (EBOV) and SARS-CoV-2, BSL-4 and BSL-3 viruses, respectively. We show that additional heat treatment, necessary for the more stringent biosafety concerns for BSL-4-derived samples, was additionally sufficient to inactivate EBOV-containing samples. Critically, this heat treatment had minimal effects on extracted RNA quality and downstream sequencing results.

## 1. Introduction

Until recently, in-depth transcriptional analysis of the immune response to viral infections was challenging due to the heterogeneity of immune cells combined with low numbers of certain cell types. Recent technical improvements have made it possible to generate RNA sequencing (RNA-seq) data using small amounts of input material down to single cell level with improved sensitivity [1,2,3,4,5]. These technical advances have been useful for studying rare immune cells [6,7] and deciphering immune responses during viral infection (reviewed in [8,9]). However, applying bulk RNA-seq protocols established for low cell numbers or single-cell sequencing (scRNA-seq) approaches to study host responses to infection with highly pathogenic viruses has been hampered by stringent biosafety requirements for sample preparation. Studies involving biosafety level 3 (BSL-3) or BSL-4 pathogens require complete virus inactivation before samples can be processed and analyzed at lower biosafety levels. Commonly used buffers to generate RNA samples in BSL-4 are TRIzol reagent and RLT buffer, which both suggest a minimal cell number of 10^5^ cells.

TCL buffer (Qiagen) is a commonly used RNA lysis buffer for the preparation of RNA-seq samples optimized for 96-well or 384-well plate applications. This enables the use of low input cell numbers and low volumes of TCL buffer (1000 cells/5 µL recommended for ultra-low input RNA sequencing) [4,5]. Comparative analysis of different lysis methods for scRNA-seq of cytotoxic T cells demonstrated optimized mRNA capture and increased detection of transcripts when using TCL buffer [1]. TCL buffer contains guanidinium isothiocyanate (GITC) which is a chaotropic reagent used to denature proteins. GITC has a virus-inactivating capacity. However, several studies have shown that the inactivation ability of GITC-containing buffers varies depending on virus load and sample type [10,11,12]. To date, there are no studies evaluating the ability of TCL buffer to inactivate viruses. Therefore, it is crucial to validate the inactivating abilities of TCL using standardized conditions, particularly for studies involving viruses that require BSL-3 or BSL-4 containment.

The cell lysis of low cell numbers in TCL is optimized for small buffer volumes. A caveat of using small volumes is that the inner surfaces of the tubes containing the samples will not be wetted with the inactivating buffer when the tubes are vortexed or shaken. However, it is important to assure that the entire content of a tube is inactivated. This can be achieved by heat inactivation of the complete sample following the chemical inactivation step in low volume.

In this study, we determined the virus inactivating ability of TCL buffer using EBOV and SARS coronavirus 2 (SARS-CoV-2) as representatives of negative and positive sense RNA viruses. EBOV belongs to the group of non-segmented negative sense RNA viruses and is classified as a BSL-4 pathogen and a Select Agent by the U. S. Department of Health and Human Services. The inactivation of EBOV-containing samples is strictly regulated in that all inactivation procedures must be validated prior to approval. For this study, we used recombinant EBOV (Mayinga isolate) expressing ZsGreen (EBOV-ZsG) [13]. EBOV-ZsG grows to high viral titers, as needed for this study, and can be easily visualized because cells infected with EBOV-ZsGreen fluoresce green.

SARS-CoV-2 is the causative agent of COVID-19 and has a positive sense RNA genome. SARS-CoV-2 has been classified as a BSL-3 agent, and complete virus inactivation is mandatory before samples may be removed from the high containment laboratory for analysis at a lower containment level.

Here, we show that TCL buffer reliably inactivates EBOV and SARS-CoV-2 in infected cells. A limited heat inactivation step was added to assure complete inactivation of the tube content. We also demonstrate that the inactivation procedures did not significantly reduce RNA quality, which is essential for downstream high-quality sequencing.

## 2. Materials and Methods

### 2.1. Biosafety Statement

All work with EBOV and SARS-CoV-2 was performed in the BSL-4 facility of Boston University’s National Emerging Infectious Diseases Laboratories (NEIDL) following approved standard operating procedures in compliance with local and national regulations pertaining to handling BSL-4 pathogens and Select Agents.

### 2.2. Cell Lines

Cell lines used in this study included African green monkey kidney cells (Vero E6; ATCC Manassas, VA, USA; CRL-1586) and human colonic epithelial cells (Caco-2; ATCC HTB-37). Vero E6 cells were maintained in Dulbecco’s modified Eagle medium (DMEM, Thermo Fisher Scientific, Waltham, MA, USA) supplemented with L-glutamine (200 mM, Thermo Fisher Scientific) and 10% fetal bovine serum (FBS; R&D Systems, Minneapolis, MN, USA). Caco-2 cells were maintained in DMEM supplemented with L-glutamine (200 mM), 1% MEM non-essential amino acids (Thermo Fisher Scientific), and 20% FBS. Cell culture media were supplemented with either penicillin (50 U/mL; Thermo Fisher Scientific) and streptomycin (50 mg/mL; Thermo Fisher Scientific) or 100 µg/mL Primocin (InvivoGen, San Diego, CA, USA). Cells were grown at 37 °C and 5% CO_2_.

### 2.3. Peripheral Blood Mononuclear Cells (PBMCs)

PMBCs from a healthy adult were purchased frozen from AllCells (Alameda, CA, USA). These experiments were performed under IRB protocols IRB-P00021163, MBG2020P000955, and IRB15-0504.

### 2.4. Coculture of PBMCs with Epithelial Cells

Cocultures of Caco-2 cells and PBMCs were generated as described previously [14]. Briefly, one day after seeding in 24-well plates (Corning, Corning, NY, USA), Caco-2 cells were washed gently twice with Caco-2 cell culture medium (see above) to remove cell debris. Frozen PBMCs were thawed in a 37 °C water bath for 90–120 s, then added dropwise to 9 mL of pre-warmed Caco-2 cell culture medium (DMEM supplemented with penicillin (50 U/mL), streptomycin (50 mg/mL), 10% FBS, 1% MEM non-essential amino acids) and centrifuged at 300× *g* for 7 min. After removing the cell supernatant, the PBMC pellet was resuspended in pre-warmed Caco-2 cell culture medium at a concentration of 1.5 × 10^6^ cells/mL. The PBMC suspension was slowly added to the epithelial cells at a final concentration of 7.5 × 10^5^ PBMCs per well. PBMCs and Caco-2 cells were then co-cultured for 14 h at 37 °C and 5% CO_2_.

### 2.5. Virus Propagation

SARS-CoV-2 stocks (isolate USA_WA1/2020, kindly provided by CDC’s Principal Investigator Natalie Thornburg and the World Reference Center for Emerging Viruses and Arboviruses (WRCEVA)) and EBOV-ZsGreen [13], were grown in Vero E6 cells cultured in DMEM supplemented with L-glutamine (200 mM), penicillin (50 U/mL), streptomycin (50 mg/mL), and 2% FBS. Virus titers were determined in Vero E6 cells by tissue culture infectious dose 50 (TCID_50_) assay using the Spearman and Kärber algorithm.

### 2.6. Amicon Column Testing

To determine virus loss during column purification, 0.5 mL of either a 2.58 × 10^9^ TCID_50_ units/mL stock of EBOV-ZsGreen or a 1.2 × 10^7^ TCID_50_ units/mL stock of SARS-CoV-2 were added to size exclusion columns (Amicon Ultra-0.5 Centrifugal Filter Unit 10 kDa; Merck, Darmstadt, Germany). Column purification was performed according to the manufacturer’s instructions. Columns were washed twice with phosphate-buffered saline (PBS, Thermo Fisher Scientific). Column content was resuspended in DMEM supplemented with L-glutamine (200 mM), penicillin (50 U/mL), streptomycin (50 mg/mL) and 2% FBS and eluted from the columns. Titers of the virus suspension eluted from the column were determined by TCID_50_ assay in comparison to non-purified viral stocks (triplicate samples each).

### 2.7. Cytotoxicity Testing

To determine successful removal of cytotoxic components from TCL buffer, TCL supplemented with 1% (*v/v*) ß-mercaptoethanol (MilliporeSigma, Burlington, MA, USA) was purified over size exclusion columns (Amicon Ultra-0.5 Centrifugal Filter Unit 10 kDa; Merck) as described above and eluted with 0.5 mL phosphate-buffered saline (PBS). As controls, non-purified TCL supplemented with ß-mercaptoethanol or PBS were used. 2 × 10^4^ VeroE6 cells seeded per well of a 96-well-plate were treated with test samples using the same ratio of column eluate to cell culture medium as used for the TCL buffer inactivation study. After incubation for 1 day, cell viability was determined using the Cell titer Glo 2.0 Assay (Promega, Madison, WI, USA) according to the manufacturer’s instructions. Each sample was prepared in 6 replicates, control cells were left untreated.

### 2.8. TCL Buffer Inactivation Testing

6 × 10^4^ to 5 × 10^5^ VeroE6 cells seeded per well of a 24-well-plate were mock-infected or infected with EBOV-ZsGreen at a multiplicity of infection (MOI) of 3 TCID_50_ units per cell or SARS-CoV-2 at an MOI of 1. Progress of the infection was monitored by analyzing ZsGreen fluorescence and/or cytopathic effects (CPE). Two (SARS-CoV-2) or four (EBOV-ZsGreen) days post-infection, when complete infection of cells or pronounced CPE was observed, cell supernatants were removed, and the cells were scraped into 0.5 mL PBS and transferred into tubes. To determine the cell number in the 24 wells at the time of inactivation, an extra well with Vero E6 cells was incubated for the same time and used to count the cells using the Luna cell counter (Logos Biosystems, Anyang, South Korea). Cell numbers varied between 3.7 × 10^5^ and 8.9 × 10^5^ cells in the various experiments.

Following the manufacturer’s instructions, TCL buffer (Qiagen, Hilden, Germany) was supplemented with ß-mercaptoethanol at a final concentration of 1% (*v/v*) and used for sample preparation the same day. Cells were pelleted by low-speed centrifugation at 500× *g* for 5 min and resuspended in 1 mL PBS or TCL. The samples were vortexed and incubated for 10 min at room temperature. All samples were purified using Amicon size exclusion columns as described above. The samples were resuspended in 0.5 mL of PBS and eluted from the columns. We used 2 columns per sample, and the eluates were combined to a total volume of 1 mL.

To ensure detection of any infectious viral particles in the samples, the entirety of each of the samples (1 mL) of the column eluates were used to infect 8 × 10^6^ Vero E6 cells seeded in T75 flasks (Thermo Fisher Scientific). For the challenge samples, EBOV-ZsGreen or SARS-CoV-2 were mixed with the column-purified eluate from TCL-treated non-infected cells and used to infect cells at an MOI of 3 (EBOV-ZsGreen) or 1 (SARS-CoV-2). Flasks were incubated for 7 days (EBOV-ZsGreen) or 4 days (SARS-CoV-2), respectively, and checked for signs of ZsGreen fluorescence and CPE every 2–3 days.

At day 7 post-infection, cell supernatants were passaged onto fresh cells to further amplify the virus. To do this, cell supernatants were clarified by low-speed centrifugation and the entire supernatant was used to infect Vero E6 cells seeded in T75 flasks. The flasks were checked for CPE every 2–3 days. At day 7 post-infection, cell supernatants were clarified by low-speed centrifugation, and 0.3 mL was used to infect Vero E6 cells seeded in a 96-well plate (Thermo Fisher Scientific). Three days post-infection, the cells were fixed with 10% formalin (LabChem, Zelienople, PA, USA).

### 2.9. Heat Inactivation Testing

To test the ability of heat to inactivate an EBOV stock solution, 1.67 × 10^6^ or 1.67 × 10^7^ TCID_50_ units of EBOV-ZsGreen in a total volume of 0.1 mL or 1 mL, respectively (cell supernatant containing EBOV particles supplemented with 10% FBS), were incubated at room temperature or at 60 °C for 30, 45, or 60 min in a Dry Block Heater (VWR 12621-088, Radnor, PA, USA). The temperature was verified by an internal heat block thermometer and an external thermometer placed in a tube in the heat block. The samples were then used to infect 8 × 10^6^ Vero E6 cells seeded in a T75 flask. Cell culture medium (DMEM supplemented with L-glutamine (200 mM), penicillin (50 U/mL), streptomycin (50 mg/mL) and 2% FBS) was used as a negative control. The cells were incubated for 7 days and checked for signs of ZsGreen fluorescence and CPE every 3–4 days. At day 7 post-infection, cell supernatants were clarified by low-speed centrifugation and the entire supernatant was used to infect Vero E6 cells seeded in T75 flasks. The flasks were checked for CPE every 3–4 days. At day 7 post-infection, cell supernatants from the second infection were clarified by low-speed centrifugation, and 0.3 mL was used to infect Vero E6 cells seeded in a 96-well plate. Three days post-infection, the cells were fixed with 10% formalin.

### 2.10. Immunofluorescence Analysis

Fixed cells were permeabilized with acetone-methanol solution (1:1; each purchased from Thermo Fisher Scientific) for 10 min at -20 °C, incubated in 0.1 M glycine (Boston BioProducts, Milford, MA, USA) for 10 min at room temperature and subsequently incubated in 5% goat serum (Jackson ImmunoResearch, West Grove, PA, USA) for 20 min at room temperature. After each step, the cells were washed three times in PBS. For SARS-CoV-2 samples, cells were incubated overnight at 4 °C with a rabbit antibody directed against the SARS-CoV nucleocapsid protein (Rockland Immunochemicals; 1:1000 dilution in 5% goat serum; Royersford, PA, USA ), which cross-reacts with the SARS-CoV-2 nucleocapsid protein [15]. For EBOV samples, cells were incubated overnight at 4 °C with a custom goat polyclonal antibody directed against the EBOV VP35 protein (Antagene; 1:200 dilution in 5% goat serum; San Jose, CA, USA). The cells were washed four times in PBS and incubated with secondary antibodies plus 4’,6-diamidino-2-phenylindole (DAPI; Sigma-Aldrich at 200 ng/mL for nuclei staining; St. Louis, MO, USA) for 1 h at room temperature. SARS-CoV-2 samples were incubated with goat anti-rabbit antibody conjugated with AlexaFluor594 (Invitrogen; 1:200 dilution in 5% goat serum; Waltham, MA, USA) and EBOV samples were incubated with donkey anti-goat antibody conjugated with AlexaFluor594 (Invitrogen; 1:200 dilution in 5% goat serum). Images were acquired using a Nikon Eclipse Ti2 microscope with Photometrics Prime BSI camera and NIS Elements AR software (Nikon, Tokyo, Japan).

### 2.11. Flow Sorting of PBMCs in Cocultures

PBMCs were harvested after 14 h of coculture, washed twice and stained using CD45 APC-H7 (clone 2D1, BD, #560178, 2:100; BD Biosciences, Franklin Lakes, NJ, USA). CD45+ cells were immediately sorted as DAPI–CD45+ on a Moflo Astrios sorter (Beckman Coulter, Brea, CA, USA). 1000 cells were sorted directly into 5 μL of TCL lysis buffer.

### 2.12. Population Low-Input RNA-seq

Low-input RNA-seq was performed following the standard ImmGen low-input protocol [5], from the 5 μL of collected lysis buffer. Smart-seq2 libraries were prepared as described previously [14]. Briefly, total RNA was captured and purified on RNAClean XP beads (Beckman Coulter). Polyadenylated mRNA was then selected using an anchored oligo(dT) primer (50–AAGCAGTGGTATCAACGCAGAGTACT30VN-30) and in vitro transcribed to generate cDNA. First strand cDNA was subjected to limited PCR amplification followed by Tn5 transposon-based fragmentation using the Nextera XT DNA Library Preparation Kit (Illumina, San Diego, CA, USA). Samples were then PCR amplified for 12 cycles using barcoded primers such that each sample carries a specific combination of eight base Illumina P5 and P7 barcodes for subsequent pooling and sequencing. Paired-end sequencing was performed on an Illumina NextSeq 500 using 2 × 38 bp reads with no further trimming.

### 2.13. RNA-seq Data Processing and QC

Reads were aligned to the human genome (GENCODE GRCh38 primary assembly and gene annotations v27) with STAR 2.5.4a (https://github.com/alexdobin/STAR/releases, accessed on 1 December 2022). The ribosomal RNA gene annotations were removed from the GTF (General Transfer Format) file. The gene-level quantification was calculated by featureCounts (http://subread.sourceforge.net/, accessed on 1 December 2022). Raw read counts tables were normalized by the median of ratios method with DESeq2 package from Bioconductor (https://bioconductor.org/packages/release/bioc/html/DESeq2.html, accessed on 1 December 2022) [16] and then converted to GCT and CLS formats.

We screened for contamination by using known cell-type-specific transcripts (per ImmGen ULI RNAseq and microarray data). Finally, the RNA integrity for all samples was measured by median Transcript Integrity Number (TIN) across human housekeeping genes with RSeQC software (http://rseqc.sourceforge.net/#tin-py, accessed on 1 December 2022). All samples had TIN <45, the usual threshold for analysis to eliminate unreliable datasets. To avoid quantitatively unreliable values from low expression, only genes with a minimum read count of 20 TPM in at least two samples were retained for analysis.

## 3. Results

### 3.1. Use of Columns to Reduce Toxicity of TCL Buffer

TCL buffer contains both GITC and N-Dodecanoyl-N-methylglycine and is supplemented with ß-mercaptoethanol shortly before use. Due to the cytotoxicity of these reagents, samples containing TCL cannot be directly assayed for the presence of infectious viruses as the treatment of cells with TCL leads to loss of cell viability (Figure S1). We used size exclusion columns (Amicon Ultra-0.5 Centrifugal Filter Unit 10 kDa) to remove cytotoxic TCL components, as demonstrated by unchanged cell viability in comparison to untreated or PBS treated cells (Figure S1)

The columns contain a cellulose-based filter that allows for the purification of products as small as 10 kilodaltons. For comparison, individual glycoproteins from EBOV and SARS-CoV-2 are approximately 100 and 180 kilodaltons, respectively, and intact viruses are much larger. Thus, these filters were predicted to efficiently retain EBOV and SARS-CoV-2, while facilitating the elimination of the much smaller components of TCL that range in size from 0.12 to 0.29 kilodaltons. Our analysis showed limited virus loss for both EBOV and SARS-CoV-2 due to purification over the Amicon columns (Figure S2), while removing the toxic components of TCL buffer (Figure S1).

### 3.2. TCL Buffer Inactivation of Ebola Virus

**Figure 1:**
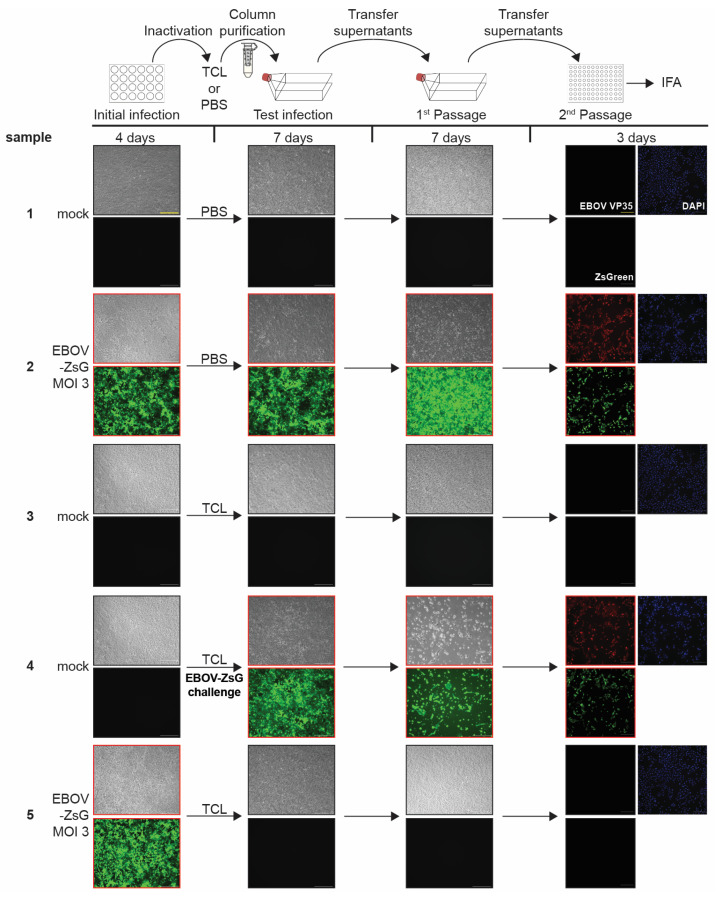
EBOV-ZsGreen inactivation by TCL. Top, schematic of the assay. Vero E6 cells seeded in 24-well plates were mock infected or infected with EBOV-ZsGreen at an MOI of 3. At 4 days post infection (dpi), brightfield and fluorescent images were taken to assess the presence of cytopathic effect (CPE) and ZsGreen expression (green fluorescence) in samples as a marker for viral infection (Initial infection). Cells were harvested in either PBS or TCL buffer, column purified, and transferred onto Vero E6 cells seeded in T75 flasks. Samples were monitored for CPE and fluorescence at 7 dpi (Test infections). Clarified supernatants were passaged onto Vero E6 cells seeded in T75 flasks. Cells were incubated for additional 7 days and monitored for viral infection (1^st^ Passage). Clarified supernatants were then used to infect Vero E6 cells seeded in 96-well plates and fixed at 3 dpi. Immunofluorescence analysis (IFA) was performed using an EBOV VP35 specific antibody. Cell nuclei were stained with DAPI. Red, EBOV VP35; green, ZsGreen fluorescence; blue, DAPI (2nd Passage). Black borders = CPE/fluorescence absent, red borders = CPE/fluorescence present. Scale bars = 250 µm. See Figure S3 for higher magnification versions of the images in this figure.

To assess whether the TCL buffer was able to inactivate EBOV, 6 × 10^4^ Vero E6 cells were mock-infected or infected with EBOV-ZsGreen at an MOI of 3. Progress of the infection was monitored by analyzing cells for CPE and fluorescence. Four days post-infection (dpi), when abundant ZsGreen expression and viral-induced CPE was observed for the EBOV-ZsGreen infected samples (Figure 1, Initial infection, samples 2 and 5, see higher magnification images in Figure S3), cells were scraped (3.7 × 10^5^ cells at time of harvest), pelleted, and resuspended in 1 mL PBS or TCL. This ratio of cell numbers to TCL volume would allow for inactivation of a maximum number of 1850 cells in 5 µL of TCL (370 cells/µL TCL) for downstream applications. The samples were vortexed, incubated for 10 min at room temperature, and purified using size exclusion columns. Initial infection rates were confirmed to be robust by ZsGreen fluorescence and IFA of replicate infections (Figure S4).

The column eluates were used to infect Vero E6 cells seeded in T75 flasks. Fluorescence and CPE were monitored over time, and the entire cell supernatant was passaged onto fresh cells at 7 dpi (Figure 1, schematic). This procedure allows even smallest amounts of infectious virus to be replicated and establish infection, enhancing the sensitivity of the assay. Passaging the entire cell supernatant onto fresh cells over a number of passages is a widely used method to show complete inactivation of viral samples [10,17,18,19]. After seven days, the passaged supernatants were used to infect cells seeded in 96-well plates, which were fixed at 3 dpi and subjected to IFA using an antibody against EBOV VP35 as an additional method of detection. Non-infected cells harvested in PBS served as a negative control (Figure 1 and Figure S3, sample 1). Incubation in PBS followed by column purification did not alter the infectivity of EBOV-ZsGreen as CPE, fluorescence, and positive staining for EBOV VP35 were observed in the respective samples (Figure 1 and Figure S3, sample 2). Column-purified lysates from mock-infected, TCL-treated cells did not induce CPE on exposed cells, indicating that the toxic components of TCL were successfully removed from the samples (Figure 1 and Figure S3, sample 3). To demonstrate that cells treated with purified TCL samples were still permissive to viral infection, infectious EBOV-ZsGreen was added to the column-purified eluate from non-infected, TCL-treated cells, and this mixture was used to infect cells (EBOV-ZsGreen challenge). Our data show that column-purified eluate did not interfere with viral infection (Figure 1 and Figure S3, sample 4). Critically, the cells infected with TCL-treated EBOV-ZsGreen did not show any signs of infection, indicating complete inactivation of EBOV-ZsGreen after incubation in TCL buffer (Figure 1 and Figure S3, sample 5). In conclusion, our data show that a 10-min incubation of 3.7 × 10^5^ infected cells in 1 mL of TCL buffer is sufficient to completely inactivate EBOV.

### 3.3. Limit of Detection Analysis for EBOV-ZsGreen

To determine the sensitivity of our testing procedure, a limit of detection analysis was performed. EBOV-ZsGreen stocks were diluted to the indicated virus amounts (1 or 10 TCID_50_ units) and used to infect Vero E6 cells seeded in T75 flasks. The cells were incubated for 7 days, and the entire clarified supernatants were passaged onto fresh Vero E6 cells seeded in T75 flasks. At seven days post-infection, the supernatants of this passage were used to infect Vero E6 cells seeded in a 96-well plate for IFA (Figure S5). While there were no signs of infection in the initial infection (Figure S5, Test infections), the limit of detection samples containing 10 TCID_50_ units, but not the sample containing 1 TCID_50_ unit, showed CPE and fluorescence and were positive for EBOV antigen after the first passage (Figure S5, 1st and 2nd Passage), indicating that that the detection assays used in this study are sufficient to reliably detect 10 infectious TCID_50_ units of EBOV. Limit of detection analysis was performed in two independent experiments.

### 3.4. TCL Buffer Inactivation of SARS-CoV-2 and Limit of Detection Study

**Figure 2:**
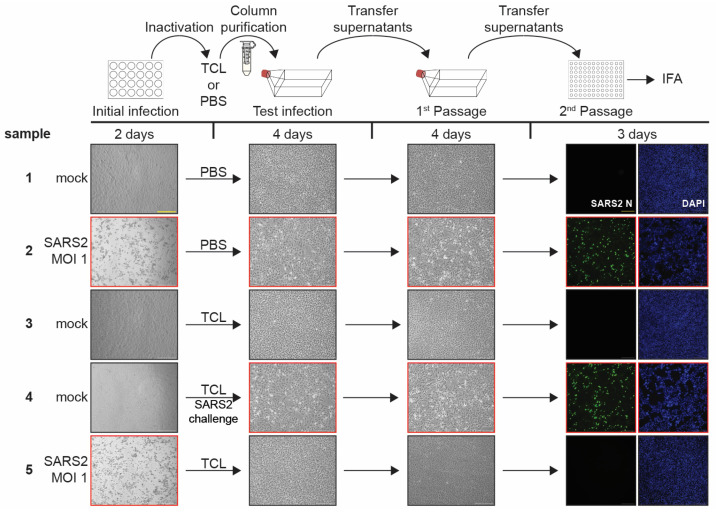
SARS-CoV-2 inactivation by TCL buffer. Top, schematic of the assay. Vero E6 cells seeded in 24-well plates were mock-infected or infected with SARS-CoV-2 at an MOI of 1. At 2 dpi, brightfield images were taken to assess the presence of CPE in samples as a marker for viral infection (Initial infection). Cells were harvested in either PBS or TCL buffer, column purified, and transferred onto Vero E6 cells seeded in T75 flasks. Samples were monitored for CPE at 4 dpi (Test infections). Clarified supernatants were passaged onto Vero E6 cells seeded in T75 flasks. The cells were incubated for additional 4 days and assessed for the presence of CPE (1^st^ Passage). Clarified supernatants were then used to infect Vero E6 cells seeded in 96-well plates and fixed at 3 dpi. Immunofluorescence analysis (IFA) was performed using a SARS-CoV-2 N specific antibody. Cell nuclei were stained with DAPI. Green, SARS-CoV-2 N; blue, DAPI (2nd Passage). Black borders = CPE/fluorescence absent, red borders = CPE/fluorescence present. Scale bars = 250 µm. See Figure S6 for higher magnification versions of the images in this figure.

In addition to EBOV, we also tested the ability of TCL to inactivate SARS-CoV-2, a positive sense RNA virus. We used the same procedures established for EBOV with some adjustments regarding the used MOI and incubation times to account for the faster replication kinetics of SARS-CoV-2 (Figure 2 and Figure S6, schematic). Initial SARS-CoV-2 infection rates were confirmed to be robust by CPE analysis and IFA using an antibody against the SARS-CoV-2 nucleoprotein (Figure 2 and Figure S6, Initial infection, and Figure S7). In the inactivation experiments, the progress of the SARS-CoV-2 infection was monitored by analyzing CPE and by IFA of final samples (Figure 2 and Figure S6, Test infections, 1st and 2nd Passage). Our data show that a 10-min incubation of 8.9 × 10^5^ infected cells in 1 mL of TCL is sufficient to completely inactivate SARS-CoV-2 (Figure 2 and Figure S6, sample 5).

Similar to the EBOV study, we also performed two independent limit of detection analyses for SARS-CoV-2. SARS-CoV-2 stocks were diluted to the indicated virus amounts (1, 10, or 100 TCID_50_ units) and used to infect Vero E6 cells seeded in T75 flasks (Figure S8). Cell supernatants were passaged twice and analyzed for infection by checking CPE and by IFA (Figure S8). We were able to reliably detect 100 infectious SARS-CoV-2 particles, whereas the virus amount in the 1 and 10 TCID_50_ unit samples was not sufficient to establish an infection.

### 3.5. Heat Inactivation of Ebola Virus

**Figure 3:**
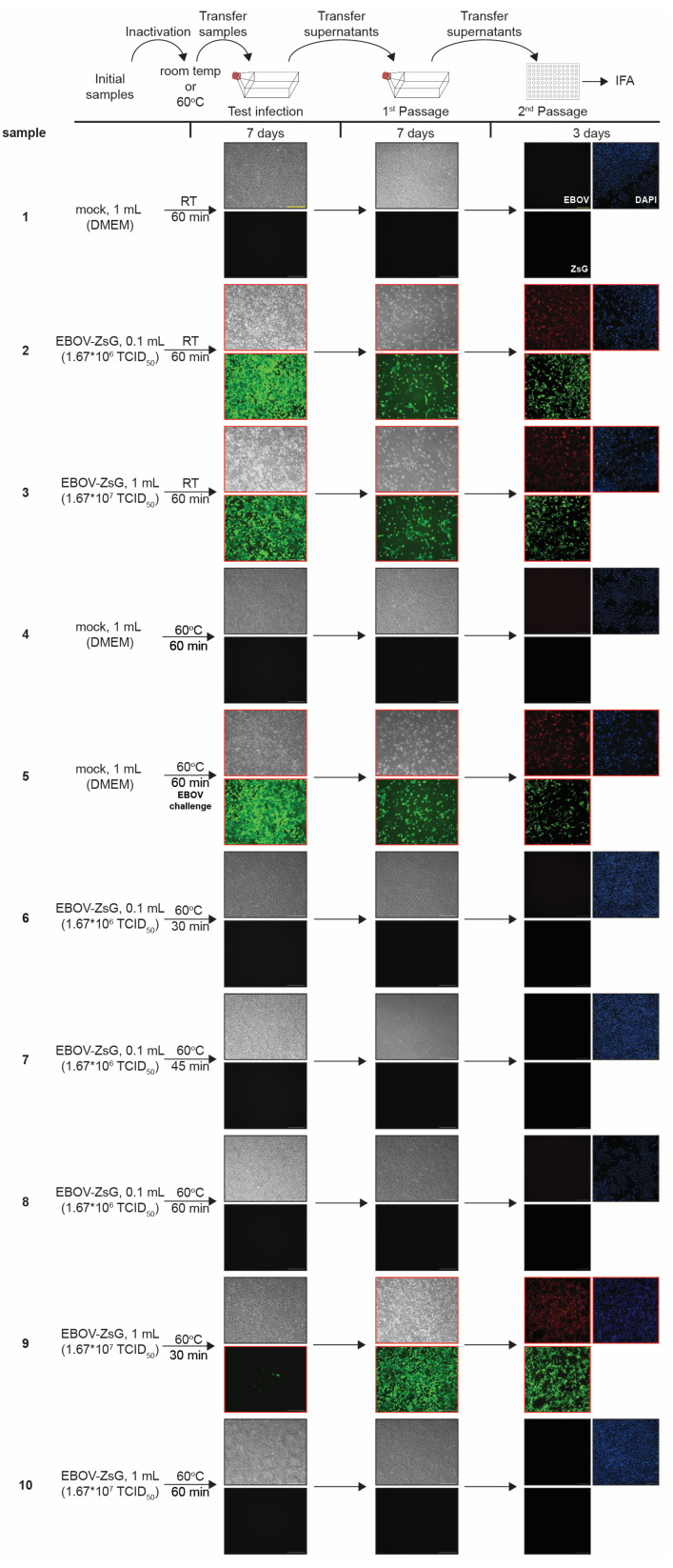
EBOV-ZsGreen inactivation by heat. Top, schematic of the assay. The indicated volumes of cell culture medium (DMEM) or EBOV-ZsGreen were incubated at room temperature (RT) or 60 °C for the indicated times and used to infect Vero E6 cells seeded in T75 flasks. At 7 dpi, brightfield and fluorescent images were taken to assess the presence of CPE and ZsGreen expression (Test infections). Clarified supernatants were transferred onto Vero E6 cells seeded in T75 flasks. The cells were incubated for additional 7 days and assessed for viral infection (1^st^ Passage). Clarified supernatants were then used to infect Vero E6 cells seeded in 96-well plates and fixed at 3 dpi. Immunofluorescence analysis (IFA) was performed using an EBOV VP35 specific antibody. Cell nuclei were stained with DAPI. Red, EBOV VP35; green, ZsGreen fluorescence; blue, DAPI (2nd Passage). Black borders = CPE/fluorescence absent, red borders = CPE/fluorescence present. Scale bars = 250 µm. See Figure S9 for higher magnification versions of the images in this figure.

Small volumes of TCL buffer are used for the optimal preparation of samples containing low cell numbers, including the generation of samples of cell types of low frequency such as specific subsets of immune cells found in peripheral blood. In our TCL inactivation studies, we used ratios of 370 (EBOV) to 890 (SARS-CoV-2) cells per *µL* TCL buffer. These small volumes of TCL buffer used to collect samples might insufficiently coat the inside of a tube during vortexing, which bears the risk of live virus potentially remaining within a tube during inactivation. Because of this biosafety concern, an additional inactivation method was added to ensure that there was no potential for live virus within small volume samples that were to be removed from BSL-4 containment. Therefore, we tested whether heat treatment at 60 °C for various times would be sufficient as an additional inactivation step. We incubated 1.67 × 10^6^ or 1.67 × 10^7^ TCID_50_ units of EBOV-ZsGreen stock solution in a total volume of 0.1 or 1 mL, respectively, at 60 °C or room temperature for 30, 45, or 60 min and used it to infect Vero E6 cells. Cell supernatants were passaged as described before. Cell culture medium was used as a negative control (Figure 3 and Figure S9, samples 1 and 4). Incubation at room temperature did not affect the ability of EBOV to infect cells using different amounts of virus stocks (Figure 3 and Figure S9, samples 2 and 3). To ensure that the cells were still permissive to infection when infected with heat-treated samples, cell culture medium incubated at 60 °C for 60 min was spiked with infectious EBOV-ZsGreen and used to infect cells (Figure 3 and Figure S9, sample 5). Our data show that each of the heat-treated EBOV-ZsGreen low volume samples (1.67 × 10^6^ infectious particles in 0.1 mL) showed no signs of infection after treatment at 60 °C for 30 min and longer (Figure 3 and Figure S9, samples 6–8). The EBOV-ZsGreen high volume sample (1.67 × 10^7^ infectious particles in 1mL) treated at 60 °C for 60 min was also completely inactivated (Figure 3 and Figure S9, sample 10). The high-volume sample treated at 60 °C for 30 min, however, showed CPE, ZsGreen fluorescence and was positive in the IFA, indicating incomplete inactivation (Figure 3 and Figure S9, sample 9). These data show that a 30-min treatment at 60 °C is sufficient to reliably inactivate lower volumes (0.1 mL) of EBOV, whereas this incubation time is insufficient for inactivating higher volumes (1 mL). In conclusion, heat inactivation at 60 °C for at least 30 min is sufficient as a supplementary method to assure complete inactivation of residual particles in chemically inactivated samples of 0.1 mL volume. In line with these results, a previous report showed successful inactivation of 140 µL samples containing up to 10^5^ infectious SARS-CoV-2 particles by incubation at 56 °C for 30 min [20], while higher viral loads led to incomplete inactivation [21].

Based on these data, our approved inactivation SOP consists of a 10-min incubation with TCL buffer at room temperature with a minimal cell number of 3.7 × 10^3^ cells in 10 µL TCL buffer and a maximal cell number of 3.7 × 10^5^ cells in 1 mL TCL buffer. After transfer of the samples into fresh tubes, this inactivation step is followed by a 45-min incubation at 60 °C to inactivate residual viral particles in low volume samples.

### 3.6. Heat Treatment Has Minor Effects on RNA Quality of Samples in TCL Buffer

**Figure 4:**
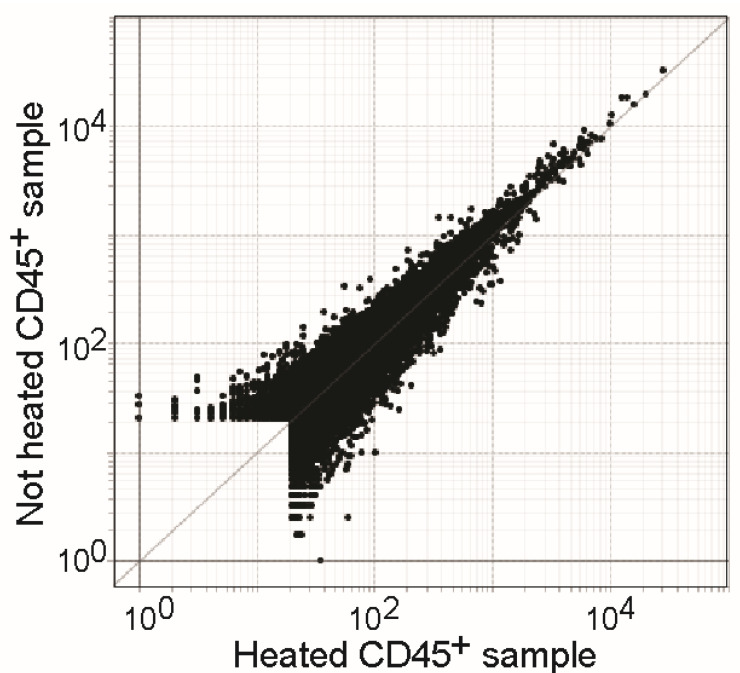
Transcriptomic effect of heat treatment on RNAseq data quality.

To assess if heat treatment of TCL-inactivated samples might lead to possibly adverse effect on RNA quality and downstream sequencing results, PBMCs were cocultured with Caco-2 cells, and CD45 positive cells were sorted by flow cytometry, lysed in TCL, and frozen at −80 °C. One day later, the samples were thawed, and half of the samples were heated at 60 °C for 45 min. Samples were then profiled by RNA sequencing (RNAseq). RNA integrity numbers (RIN) were not significantly impacted by the heat treatment (53.1 *±* 4.0 in heated versus 59.2 *±* 1.9 in unheated samples, *p* = 0.23), and neither were the read counts. Overall, the transcriptomic results were highly similar in the two settings, as shown in the expression-expression plot (Figure 4).Using the above described inactivation (TCL lysis and heat at 60 °C), we successfully analyzed transcriptomic responses of different PBMC subsets after coculture with infected Caco-2 cells (SARS-CoV-2 or EBOV) using ultra low input RNA sequencing [14].

Expression-Expression plot of heated (x-axis) versus non-heated (y-axis) RNA from CD45+ cells in a coculture setting.

## 4. Discussion

This study aimed to validate a TCL-based inactivation method for EBOV- and SARS-CoV-2-infected cells. TCL buffer is used for RNA preparation of low cell numbers, including single cells. In our inactivation studies, we demonstrated that a ratio of 370–890 cells per µL of TCL buffer resulted in complete inactivation of EBOV and SARS-COV-2. This ratio was selected as it aligns with established cell-to-TCL ratios for low input RNA sequencing (1000 cells per 5 µL TCL = 200 cells per µL TCL [5]. Similar to other common RNA lysis buffers, TCL contains GITC, a chaotropic, protein denaturing reagent. Previous inactivation studies with GITC-containing buffers showed complete inactivation in some reports [22] and incomplete inactivation in others [10,11,12]. A possible explanation for these discrepancies could be differences in the ratio of cell numbers or infectious viral particles to RNA lysis buffer volume or differences in the concentration of the inactivating components in the buffers. In addition, it is possible that some viruses are easier to inactivate than others. For example, complete inactivation of 2.5 × 10^6^ cells infected with H5N1 influenza A virus was achieved after incubation in 1 mL RLT buffer (Qiagen) [22], whereas 5 × 10^6^ EBOV-infected cells were not completely inactivated after incubation in 600 and 800 µL RLT, respectively [10]. This shows that the specific conditions of inactivation must be carefully determined, including virus species, cell numbers/infectious viral particles, lysis buffer volume, and incubation times. Besides RLT, TRIzol is also commonly used for virus inactivation in high containment settings [10,17,19,23,24,25,26,27], yet neither of these buffers is recommended for use with low cell numbers unlike TCL, which can be used even at the single cell level [1,2]. Our analysis showed limited virus loss for both EBOV and SARS-CoV-2 due to purification over the Amicon columns to remove cytotoxic components (Figure S2), similar to previously described SARS-CoV-2 yields using these columns [28]. Due to the low volume of samples generated, we included heat treatment as an additional inactivation step. Importantly, full inactivation of 1 mL samples containing 1.67 × 10^7^ infectious particles EBOV was only observed at 60 min of heat treatment at 60 °C, while shorter incubation times still yielded infectious virus (Figure 3 and Figure S9). Similar observations have been made by others, with varying temperatures and times needed for complete virus inactivation, as well as variability depending on the virus and other sample parameters tested (for example, sample volumes or the presence or absence of FBS) [10,21,29,30,31,32,33]. This highlights the need for careful validation of inactivation procedures and resulting standard operating procedures to ensure complete inactivation of viral samples. In this instance, the tested heat procedure is used as a supplementary safety measure in addition to the use of TCL which is effective in inactivating virus. Importantly, the extra heat step had no negative influence on RNA quality in terms of downstream sequencing. We successfully document inactivation of both negative sense (EBOV) and positive sense RNA viruses (SARS-CoV-2) using TCL, enabling the transfer of TCL lysates to lower biosafety levels and use of high-quality sequencing equipment which most times is not available inside containment laboratories. It should be noted that the genomes of positive sense RNA viruses can be used to transfect cells to generate infectious progeny virus. For this reason, inactivated samples of positive sense RNA viruses that are classified as Select Agents, such as Venezuelan equine encephalitis virus and SARS-CoV-1, are also classified as Select Agents as long as the RNA genome is intact [34]. However, rescue of SARS-CoV-2 (not a Select Agent) from RNA genomes requires the addition of plasmid-encoded nucleoprotein to be efficient [35]. Therefore, the risk of misuse of TCL-inactivated samples is considered very low.

## Data Availability

The data reported in this paper have been deposited in the Gene Expression Omnibus database under accession GSE186650.

## Supplementary Materials

The following supporting information can be downloaded at: https://www.mdpi.com/article/10.3390/pathogens12020342/s1, Figure S1: Reduction in cytotoxicity of TCL buffer using column purification; Figure S2: Effect of column purification on virus recovery; Figure S3: Higher resolution versions of images from Figure 1; Figure S4: Initial infection rates of Vero E6 cells infected with EBOV-ZsGreen; Figure S5: EBOV-ZsGreen limit of detection analysis; Figure S6: Higher resolution versions of images from Figure 2; Figure S7: Initial infection rates of Vero E6 cells infected with SARS-CoV-2; Figure S8: SARS-CoV-2 limit of detection analysis; Figure S9: Higher resolution versions of images from Figure 3.

## References

[1] Kanev K., Roelli P., Wu M., Wurmser C., Delorenzi M., Pfaffl M.W., Zehn D. (2021). Tailoring the resolution of single-cell RNA sequencing for primary cytotoxic T cells. Nat. Commun..

[2] Picelli S. (2019). Full-Length Single-Cell RNA Sequencing with Smart-seq2. Methods Mol. Biol..

[3] Picelli S., Faridani O.R., Bjorklund A.K., Winberg G., Sagasser S., Sandberg R. (2014). Full-length RNA-seq from single cells using Smart-seq2. Nat. Protoc..

[4] Trombetta J.J., Gennert D., Lu D., Satija R., Shalek A.K., Regev A. (2014). Preparation of Single-Cell RNA-Seq Libraries for Next Generation Sequencing. Curr. Protoc. Mol. Biol..

[5] Immunological Genome Project Ultra-Low-Input RNA-Seq (ULI RNA-Seq). https://www.immgen.org/Protocols/ImmGenULI_RNAseq_methods.pdf.

[6] Reyes M., Vickers D., Billman K., Eisenhaure T., Hoover P., Browne E.P., Rao D.A., Hacohen N., Blainey P.C. (2019). Multiplexed enrichment and genomic profiling of peripheral blood cells reveal subset-specific immune signatures. Sci. Adv..

[7] Lam V.C., Folkersen L., Aguilar O.A., Lanier L.L. (2019). KLF12 Regulates Mouse NK Cell Proliferation. J. Immunol..

[8] Luo G., Gao Q., Zhang S., Yan B. (2020). Probing infectious disease by single-cell RNA sequencing: Progresses and perspectives. Comput. Struct. Biotechnol. J..

[9] Rato S., Golumbeanu M., Telenti A., Ciuffi A. (2017). Exploring viral infection using single-cell sequencing. Virus Res..

[10] Haddock E., Feldmann F., Feldmann H. (2016). Effective Chemical Inactivation of Ebola Virus. Emerg. Infect. Dis..

[11] Ngo K.A., Jones S.A., Church T.M., Fuschino M.E., George K.S., Lamson D.M., Maffei J., Kramer L.D., Ciota A.T. (2017). Unreliable Inactivation of Viruses by Commonly Used Lysis Buffers. Appl. Biosaf..

[12] Smither S.J., Weller S.A., Phelps A., Eastaugh L., Ngugi S., O’Brien L.M., Steward J., Lonsdale S.G., Lever M.S. (2015). Buffer AVL Alone Does Not Inactivate Ebola Virus in a Representative Clinical Sample Type. J. Clin. Microbiol..

[13] Hume A.J., Heiden B., Olejnik J., Suder E.L., Ross S., Scoon W.A., Bullitt E., Ericsson M., White M.R., Turcinovic J. (2022). Recombinant Lloviu virus as a tool to study viral replication and host responses. PLoS Pathog..

[14] Leon J., Michelson D.A., Olejnik J., Chowdhary K., Oh H.S., Hume A.J., Galvan-Pena S., Zhu Y., Chen F., Vijaykumar B. (2022). A virus-specific monocyte inflammatory phenotype is induced by SARS-CoV-2 at the immune-epithelial interface. Proc. Natl. Acad. Sci. USA.

[15] Huang J., Hume A.J., Abo K.M., Werder R.B., Villacorta-Martin C., Alysandratos K.D., Beermann M.L., Simone-Roach C., Lindstrom-Vautrin J., Olejnik J. (2020). SARS-CoV-2 Infection of Pluripotent Stem Cell-Derived Human Lung Alveolar Type 2 Cells Elicits a Rapid Epithelial-Intrinsic Inflammatory Response. Cell Stem Cell.

[16] Love M.I., Huber W., Anders S. (2014). Moderated estimation of fold change and dispersion for RNA-seq data with DESeq2. Genome Biol..

[17] Alfson K.J., Griffiths A. (2018). Development and Testing of a Method for Validating Chemical Inactivation of Ebola Virus. Viruses.

[18] Jureka A.S., Silvas J.A., Basler C.F. (2020). Propagation, Inactivation, and Safety Testing of SARS-CoV-2. Viruses.

[19] Kochel T.J., Kocher G.A., Ksiazek T.G., Burans J.P. (2017). Evaluation of TRIzol LS Inactivation of Viruses. Appl. Biosaf..

[20] Auerswald H., Yann S., Dul S., In S., Dussart P., Martin N.J., Karlsson E.A., Garcia-Rivera J.A. (2021). Assessment of inactivation procedures for SARS-CoV-2. J. Gen. Virol..

[21] Pastorino B., Touret F., Gilles M., de Lamballerie X., Charrel R.N. (2020). Heat Inactivation of Different Types of SARS-CoV-2 Samples: What Protocols for Biosafety, Molecular Detection and Serological Diagnostics?. Viruses.

[22] Avelin V., Sissonen S., Julkunen I., Osterlund P. (2022). Inactivation efficacy of H5N1 avian influenza virus by commonly used sample preparation reagents for safe laboratory practices. J. Virol. Methods.

[23] Blow J.A., Dohm D.J., Negley D.L., Mores C.N. (2004). Virus inactivation by nucleic acid extraction reagents. J. Virol. Methods.

[24] Jensen K.S., Adams R., Bennett R.S., Bernbaum J., Jahrling P.B., Holbrook M.R. (2018). Development of a novel real-time polymerase chain reaction assay for the quantitative detection of Nipah virus replicative viral RNA. PLoS ONE.

[25] Widerspick L., Vazquez C.A., Niemetz L., Heung M., Olal C., Bencsik A., Henkel C., Pfister A., Brunetti J.E., Kucinskaite-Kodze I. (2022). Inactivation Methods for Experimental Nipah Virus Infection. Viruses.

[26] Lo M.K., Miller D., Aljofan M., Mungall B.A., Rollin P.E., Bellini W.J., Rota P.A. (2010). Characterization of the antiviral and inflammatory responses against Nipah virus in endothelial cells and neurons. Virology.

[27] Mire C.E., Satterfield B.A., Geisbert J.B., Agans K.N., Borisevich V., Yan L., Chan Y.P., Cross R.W., Fenton K.A., Broder C.C. (2016). Pathogenic Differences between Nipah Virus Bangladesh and Malaysia Strains in Primates: Implications for Antibody Therapy. Sci. Rep..

[28] Welch S.R., Davies K.A., Buczkowski H., Hettiarachchi N., Green N., Arnold U., Jones M., Hannah M.J., Evans R., Burton C. (2020). Analysis of Inactivation of SARS-CoV-2 by Specimen Transport Media, Nucleic Acid Extraction Reagents, Detergents, and Fixatives. J. Clin. Microbiol..

[29] Mitchell S.W., McCormick J.B. (1984). Physicochemical inactivation of Lassa, Ebola, and Marburg viruses and effect on clinical laboratory analyses. J. Clin. Microbiol..

[30] Bowen E.T., Simpson D.I., Bright W.F., Zlotnik I., Howard D.M. (1969). Vervet monkey disease: Studies on some physical and chemical properties of the causative agent. Br. J. Exp. Pathol..

[31] Patterson E.I., Prince T., Anderson E.R., Casas-Sanchez A., Smith S.L., Cansado-Utrilla C., Solomon T., Griffiths M.J., Acosta-Serrano A., Turtle L. (2020). Methods of Inactivation of SARS-CoV-2 for Downstream Biological Assays. J. Infect. Dis..

[32] Rabenau H.F., Cinatl J., Morgenstern B., Bauer G., Preiser W., Doerr H.W. (2005). Stability and inactivation of SARS coronavirus. Med. Microbiol. Immunol..

[33] Darnell M.E., Subbarao K., Feinstone S.M., Taylor D.R. (2004). Inactivation of the coronavirus that induces severe acute respiratory syndrome, SARS-CoV. J. Virol. Methods.

[34] Behnia M., Baer A., Skidmore A.M., Lehman C.W., Bracci N., Kehn-Hall K., Bradfute S.B. (2022). Inactivation of Venezuelan Equine Encephalitis Virus Genome Using Two Methods. Viruses.

[35] Thi Nhu Thao T., Labroussaa F., Ebert N., V’Kovski P., Stalder H., Portmann J., Kelly J., Steiner S., Holwerda M., Kratzel A. (2020). Rapid reconstruction of SARS-CoV-2 using a synthetic genomics platform. Nature.

