# Supplementary material for: Establishment of an Inactivation Method for Ebola Virus and SARS-CoV-2 Suitable for Downstream Sequencing of Low Cell Numbers
Source: Pathogens. 2023 Feb 17;12(2):342. doi: 10.3390/pathogens12020342 (PMC9958562; doi:10.3390/pathogens12020342)
Supplement: Supplementary file 1 [file pathogens-12-00342-s001.zip › pathogens-2133226-supplementary.pdf]

Article

Establishment of an Inactivation Method for Ebola Virus and SARS-CoV-2 Suitable for Downstream Sequencing of Low Cell Numbers

Judith Olejnik [1,2], Juliette Leon [3,4], Daniel Michelson [3], Kaitavjeet Chowdhary [3], Silvia Galvan-Pena [3], Christophe Benoist [3], Elke Mühlberger [1,2,*] and Adam J Hume [1,2,5,*]

[1] Department of Microbiology, Boston University School of Medicine, Boston, MA 02118, USA
[2] National Emerging Infectious Diseases Laboratories, Boston University, Boston, MA 02218, USA
[3] Department of Immunology, Blavatnik Institute, Harvard Medical School, Boston, MA 02115, USA
[4] INSERM UMR 1163, Institut Imagine, University of Paris, 75015 Paris, France
[5] Center for Emerging Infectious Diseases Policy & Research, Boston University, Boston, MA 02118, USA
* Correspondence: muehlber@bu.edu (E.M.); hume@bu.edu (A.J.H.); Tel.: +1-617-358-9153 (E.M.); +1-617-358-9151 (A.J.H.)

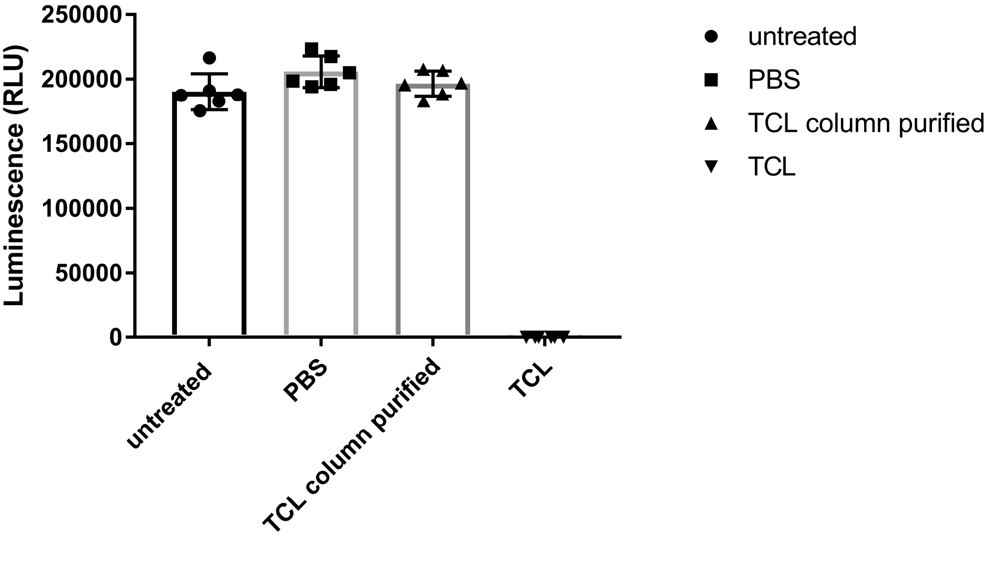

Figure S1. Reduction in cytotoxicity of TCL buffer using column purification. Vero E6 cells were left untreated or incubated with PBS, TCL supplemented with ß-mercaptoethanol at a final concentration of 1% (v/v), or column purified TCL containing 1% ß-mercaptoethanol using the same ratio of sample to cell culture medium (v/v) as used for the TCL buffer inactivation testing. After incubation for 1 day, cell viability was determined. Each sample was prepared in 6 replicates, control cells were left untreated.

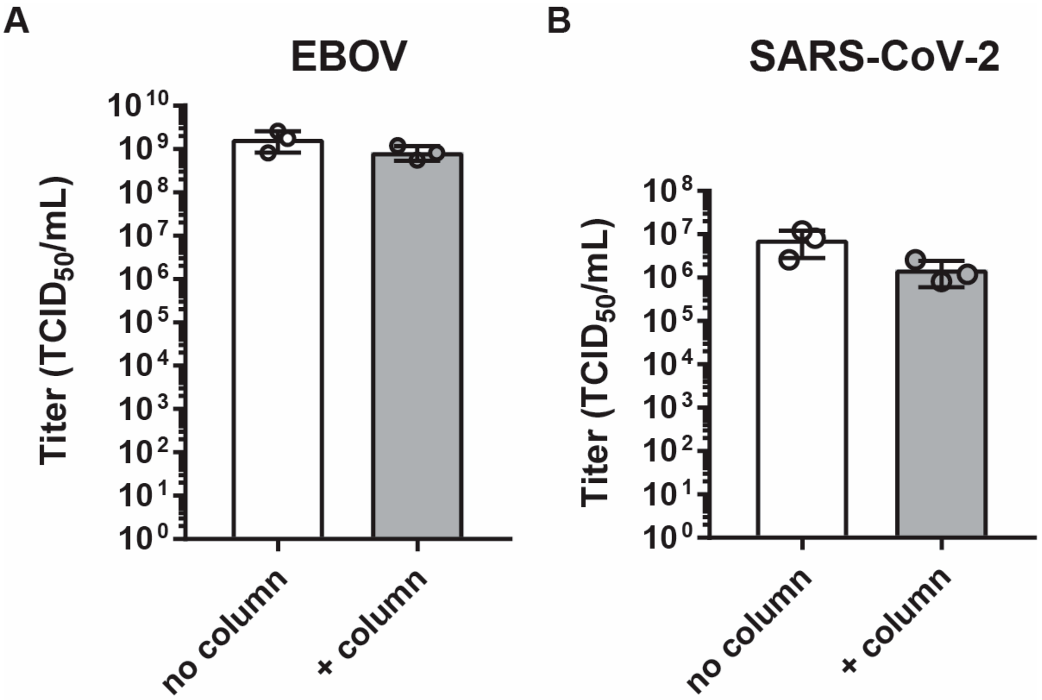

Figure S2. Effect of column purification on virus recovery. 0.5 mL of either a 2.58×10^9 $TCID_{50}$ units/mL stock of EBOV-ZsGreen or a 1.2×10^7 $TCID_{50}$ units/mL stock of SARS-CoV-2 were added to size exclusion columns. Column purification was performed according to the manufacturer's instructions. Viral titers of samples eluted from the column and non-column purified virus stock were determined by $TCID_{50}$ assay.

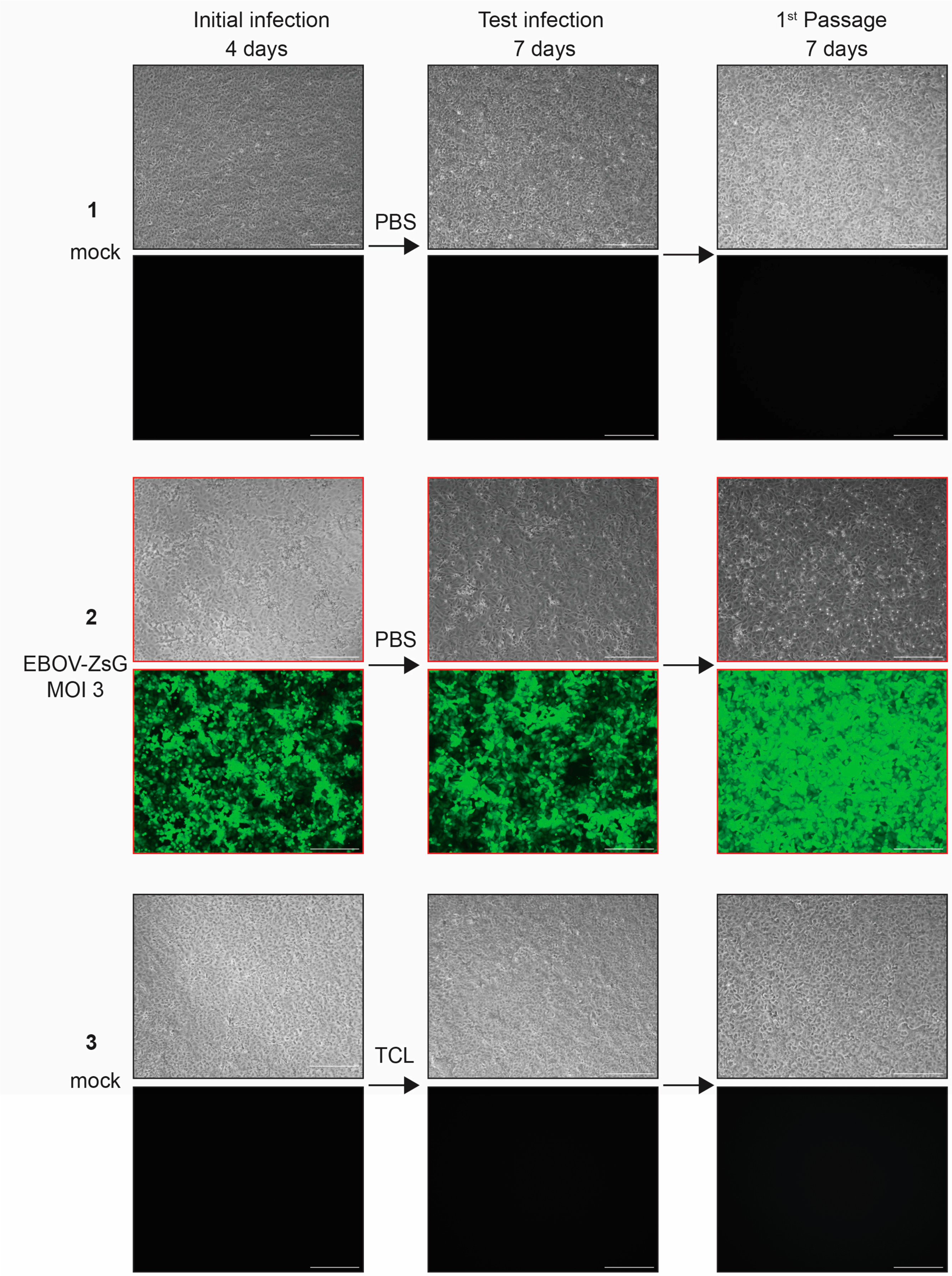

Figure S3. (1/3) Higher resolution versions of images from Figure 1. Scale bars = 250 μm.

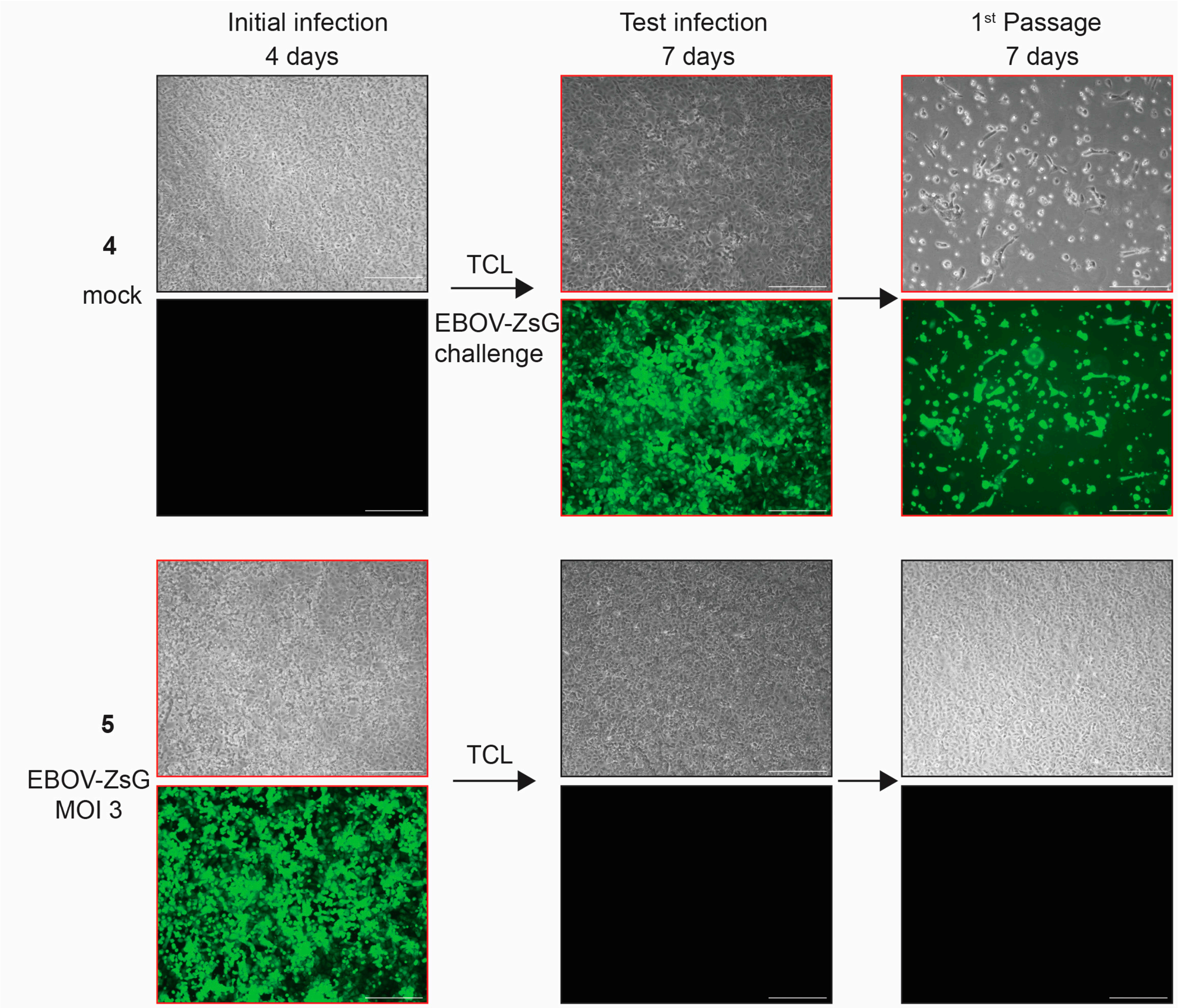

Figure S3. (2/3) Higher resolution versions of images from Figure 1. Scale bars = 250 µm.

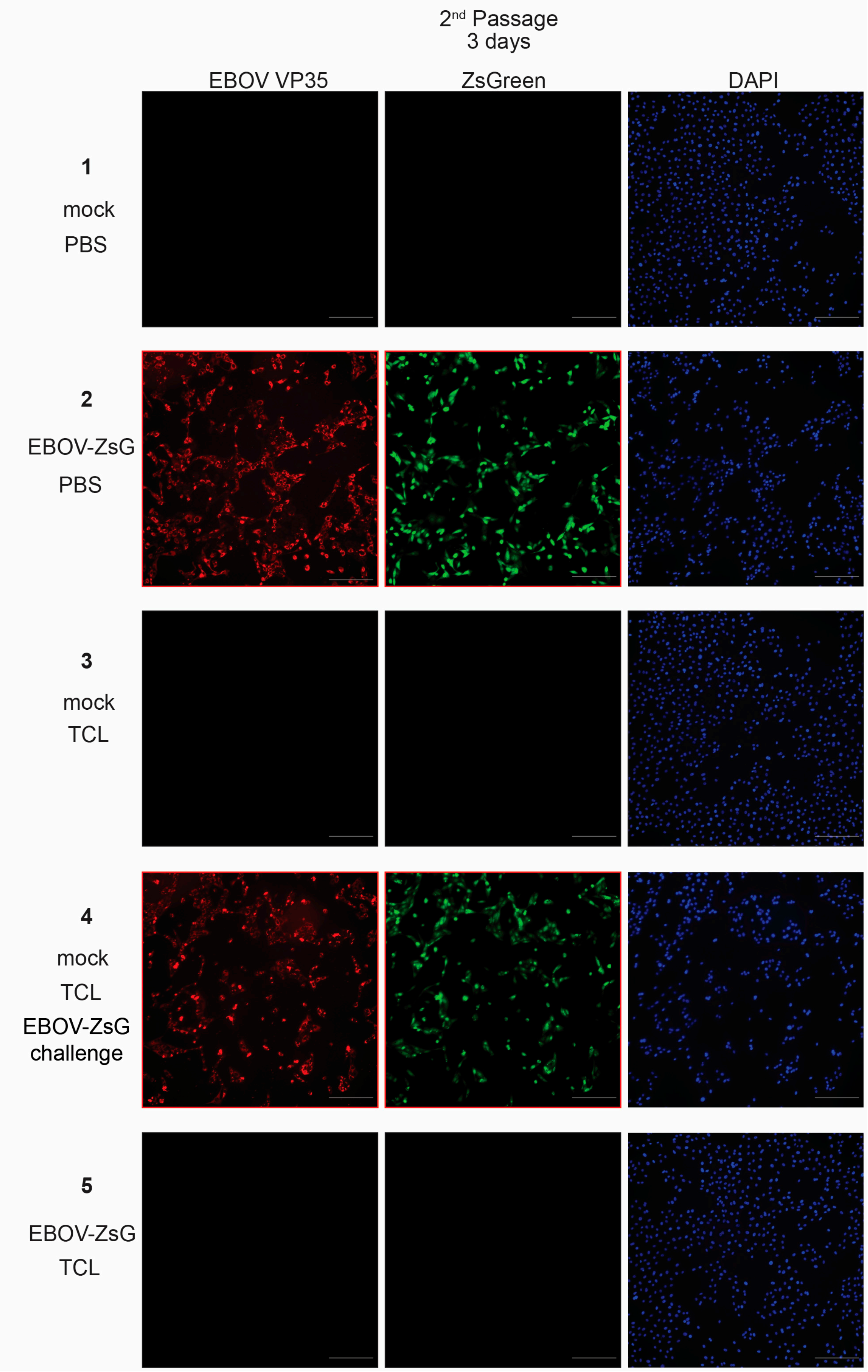

Figure S3. (3/3) Higher resolution versions of images from Figure 1. Scale bars = 250 µm.

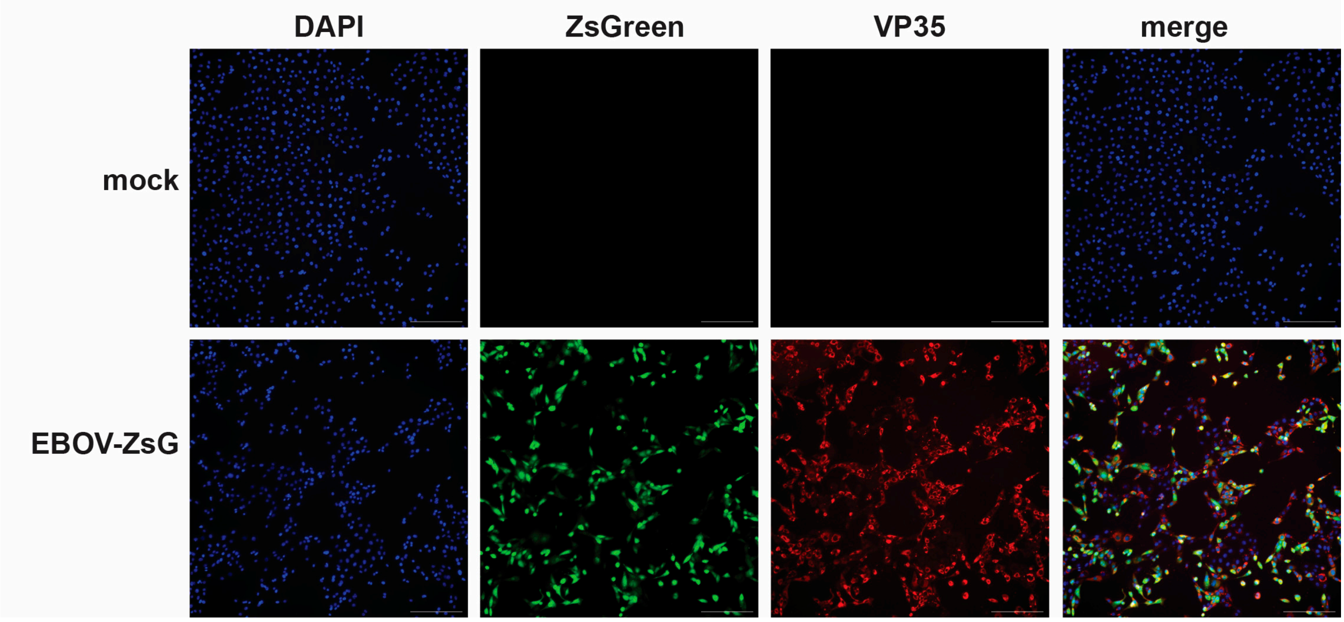

Figure S4. Initial infection rates of Vero E6 cells infected with EBOV-ZsGreen. Vero E6 cells seeded in 8-well chamber slides were mock-infected or infected with EBOV-ZsGreen (MOI = 3) in parallel with the infections in Figures 1 and S3 and fixed at 4 dpi. Immunofluorescence analysis was performed using an EBOV VP35 specific antibody. Cell nuclei were stained with DAPI. Red, EBOV VP35; green, ZsGreen fluorescence; blue, DAPI. Scale bars = 250 μm.

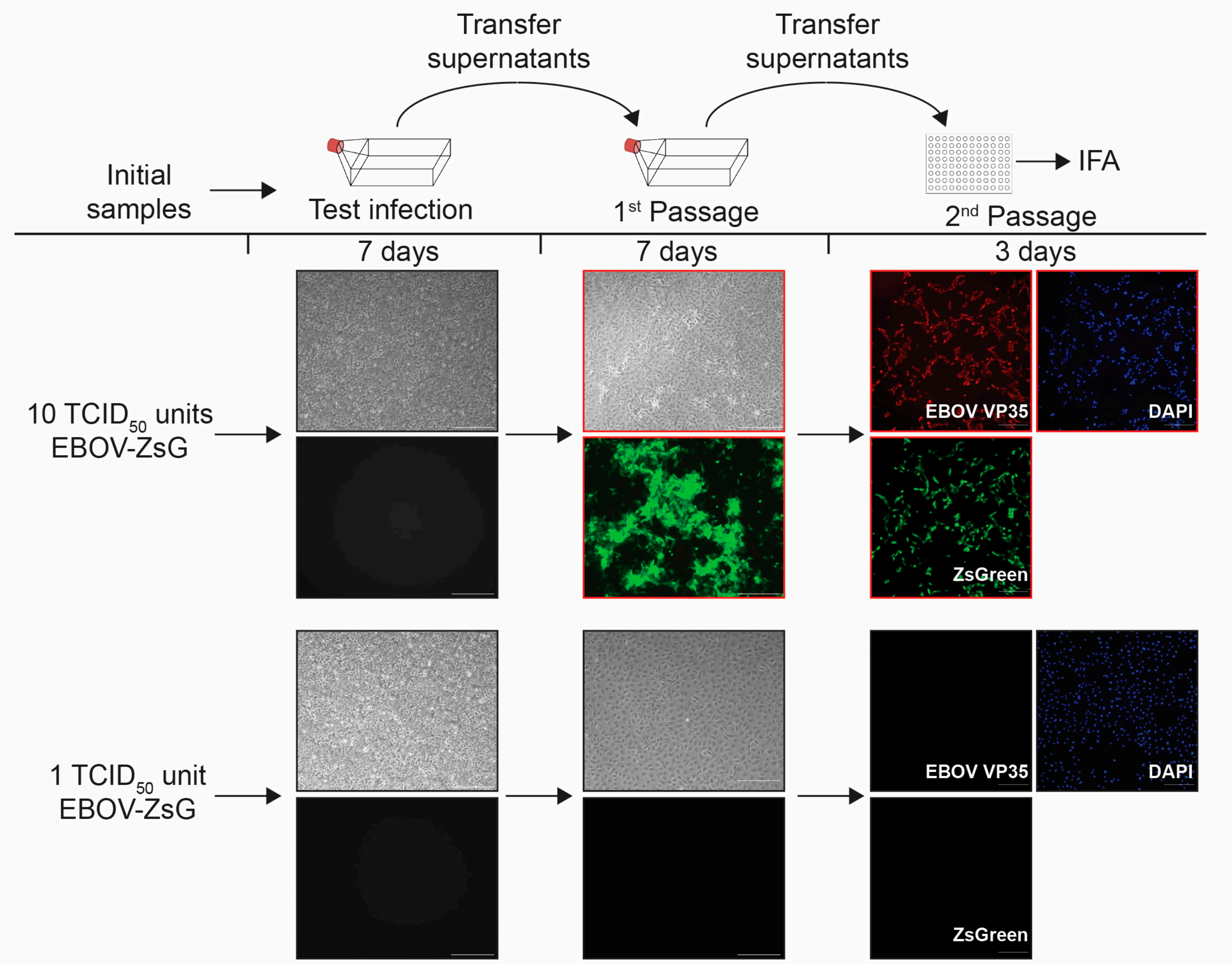

Figure S5. EBOV-ZsGreen limit of detection analysis. Top, schematic of the assay. Vero E6 cells seeded in T75 flasks were infected with the indicated amounts of infectious EBOV-ZsGreen particles. Samples were assessed for CPE and ZsGreen expression at 7dpi (Test infections). Clarified supernatants were used to infect Vero E6 cells seeded in T75 flasks. At 7 dpi, cells were assessed for the presence of CPE and fluorescence (1st Passage). Clarified supernatants were then used to infect Vero E6 cells seeded in 96-well plates and fixed at 3 dpi. Immunofluorescence analysis (IFA) was performed using an EBOV VP35 specific antibody. Cell nuclei were stained with DAPI. Red, EBOV VP35; green, ZsGreen fluorescence; blue, DAPI (2nd Passage). Black borders = CPE/fluorescence absent, red borders = CPE/fluorescence present. Scale bars = 250 µm.

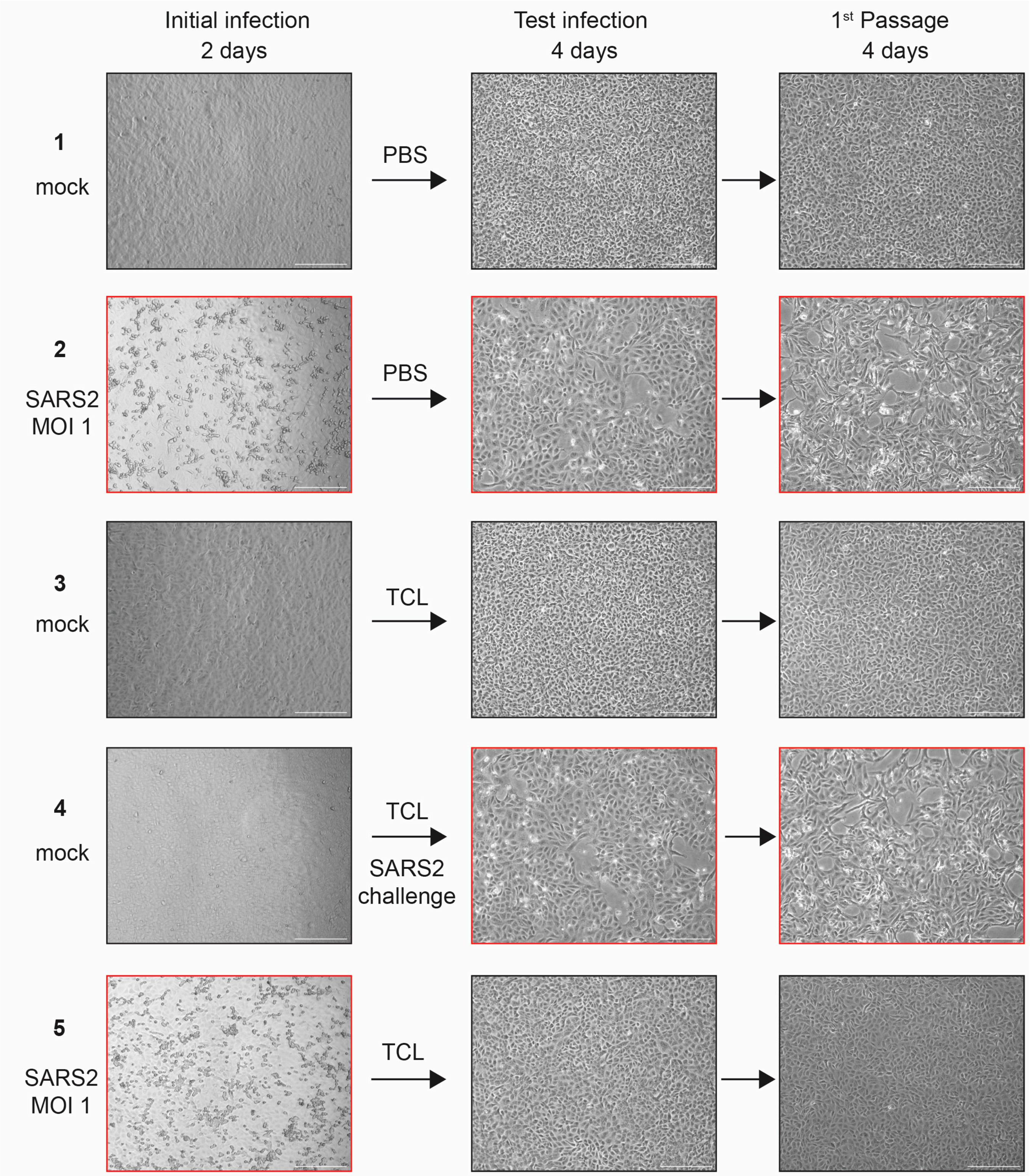

Figure S6. (1/2) Higher resolution versions of images from Figure 2. Scale bars = 250 µm.

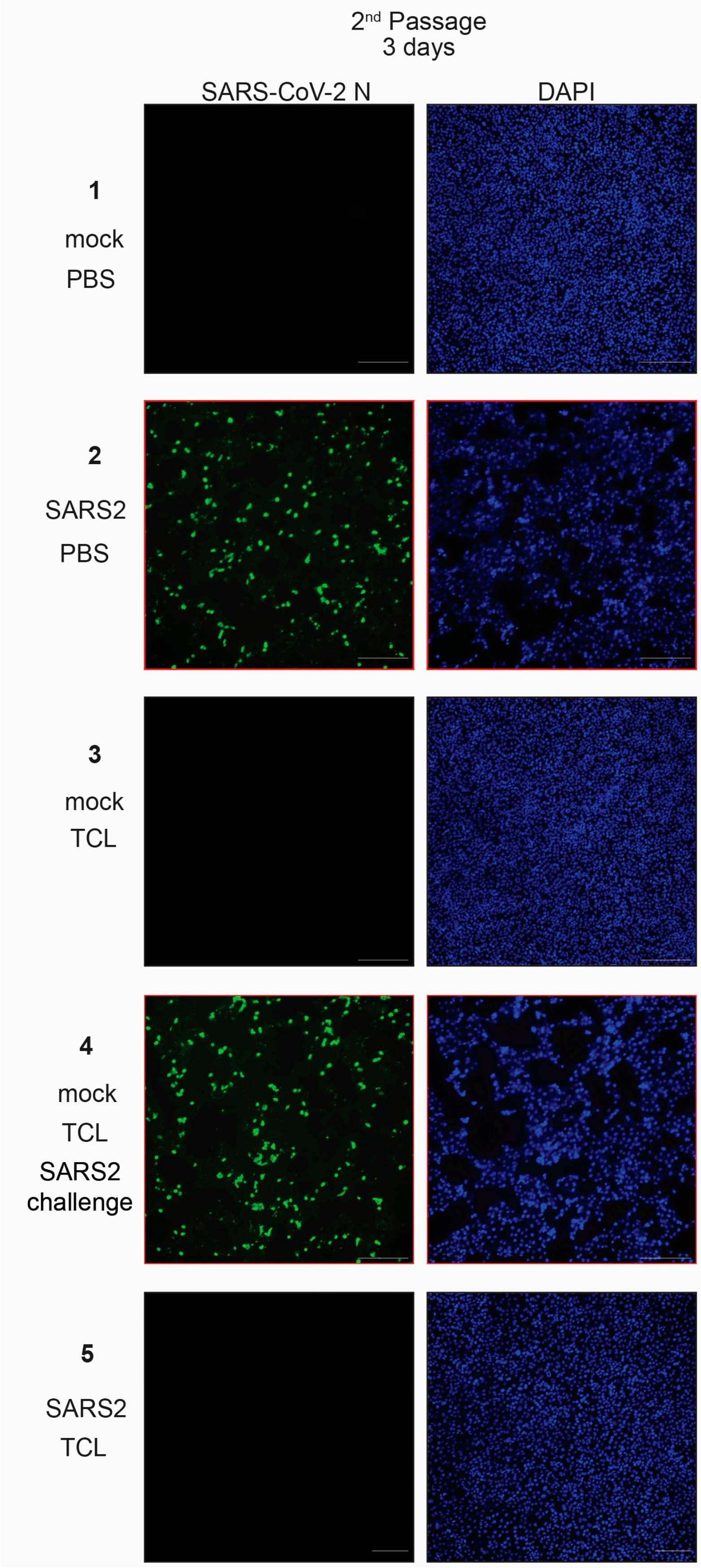

Figure S6. (2/2) Higher resolution versions of images from Figure 2. Scale bars = 250 μm.

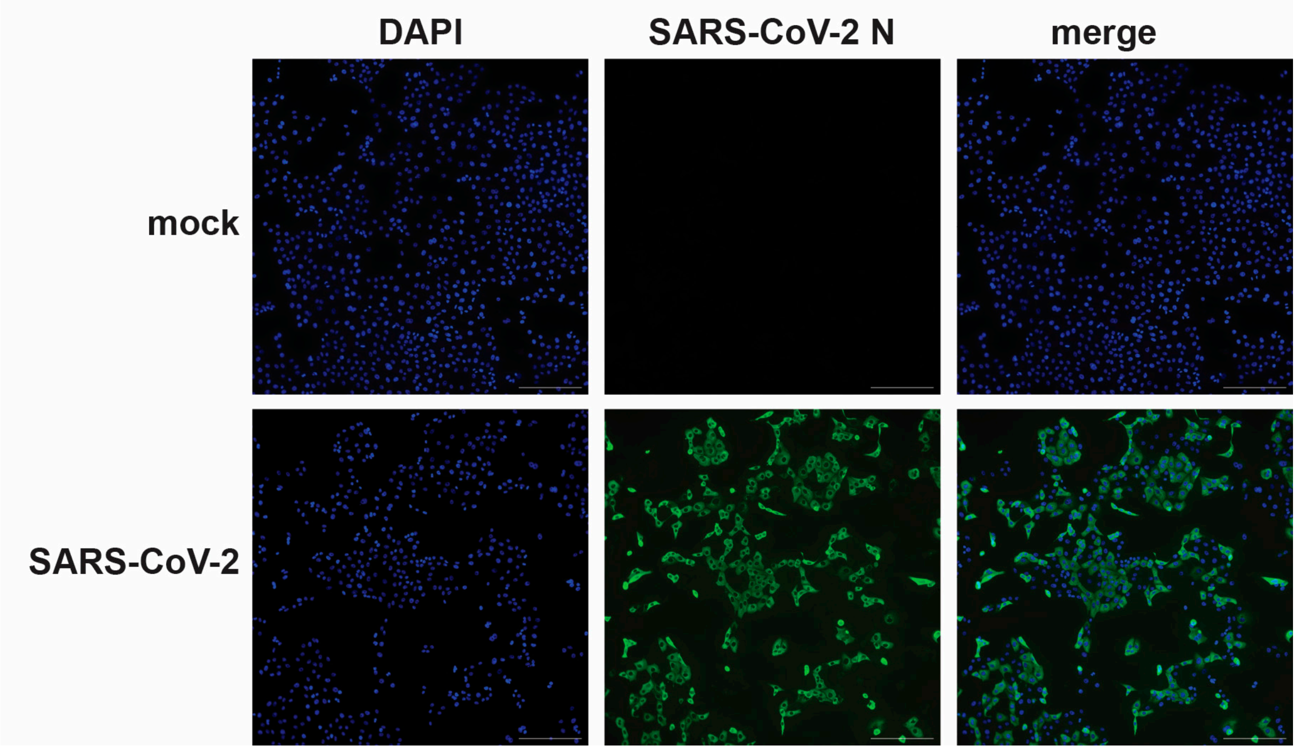

Figure S7. Initial infection rates of Vero E6 cells infected with SARS-CoV-2. Vero E6 cells seeded in 8-well chamber plates were mock-infected or infected with SARS-CoV-2 (MOI = 1) in parallel with the infections in Figures 2 and S6 and fixed at 2 dpi. Immunofluorescence analysis was performed using a SARS-CoV-2 N specific antibody. Cell nuclei were stained with DAPI. Green, SARS-CoV-2 N; blue, DAPI. Scale bars = 250 µm.

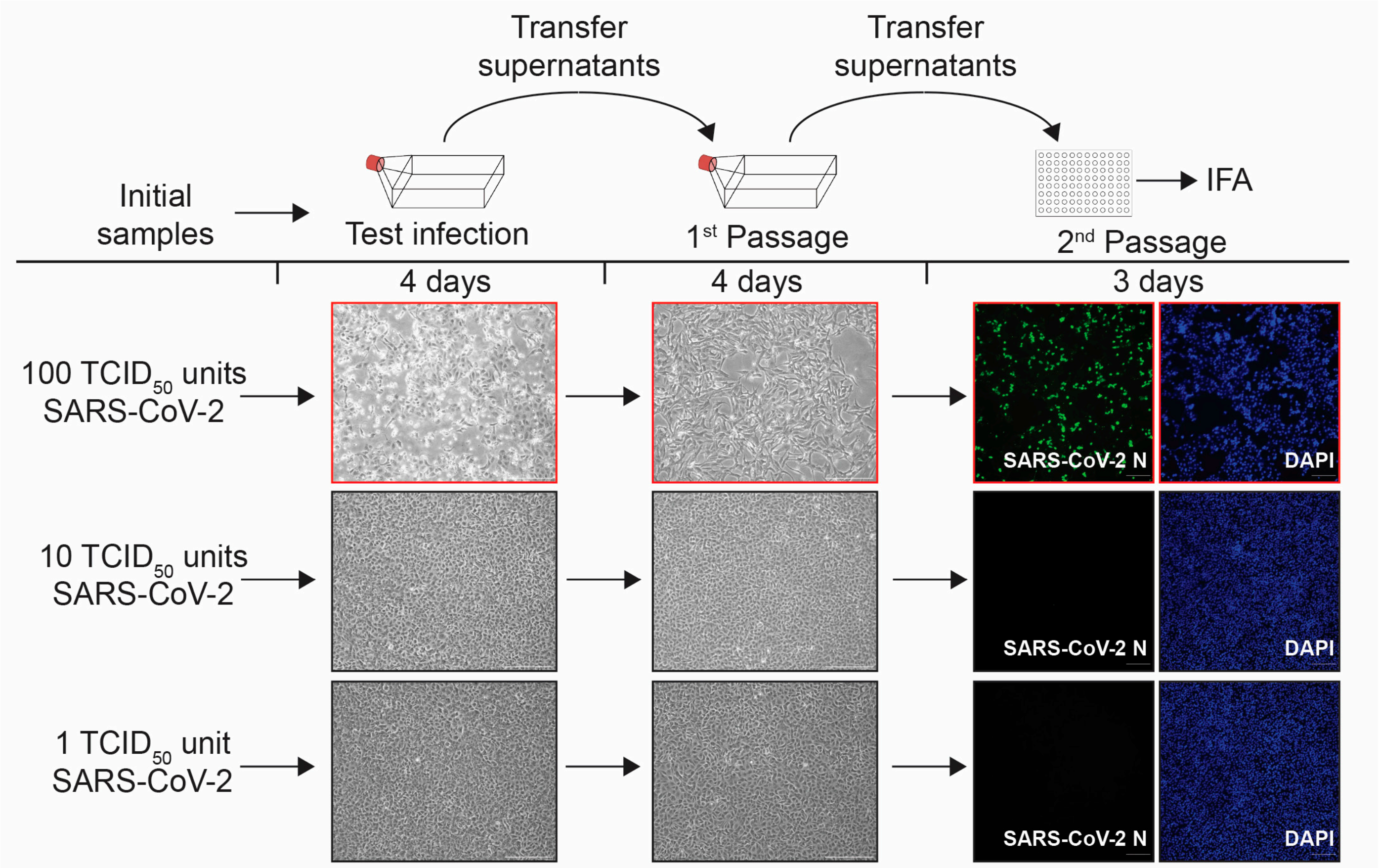

Figure S8. SARS-CoV-2 limit of detection analysis. Top, schematic of the assay. Vero E6 cells seeded in T75 flasks were infected with the indicated amounts of infectious SARS-CoV-2 particles. Samples were assessed for CPE at 4 dpi (Test infections). Clarified supernatants were transferred onto Vero E6 cells. At 4 dpi, cells were assessed for the presence of CPE (1st Passage). Clarified supernatants were then used to infect Vero E6 cells seeded in 96-well plates and fixed at 3 dpi. Immunofluorescence analysis (IFA) was performed using a SARS-CoV-2 N specific antibody. Cell nuclei were stained with DAPI. Green, SARS-CoV-2 N; blue, DAPI (2nd Passage). Black borders = CPE absent, red borders = CPE present. Scale bars = 250 µm.

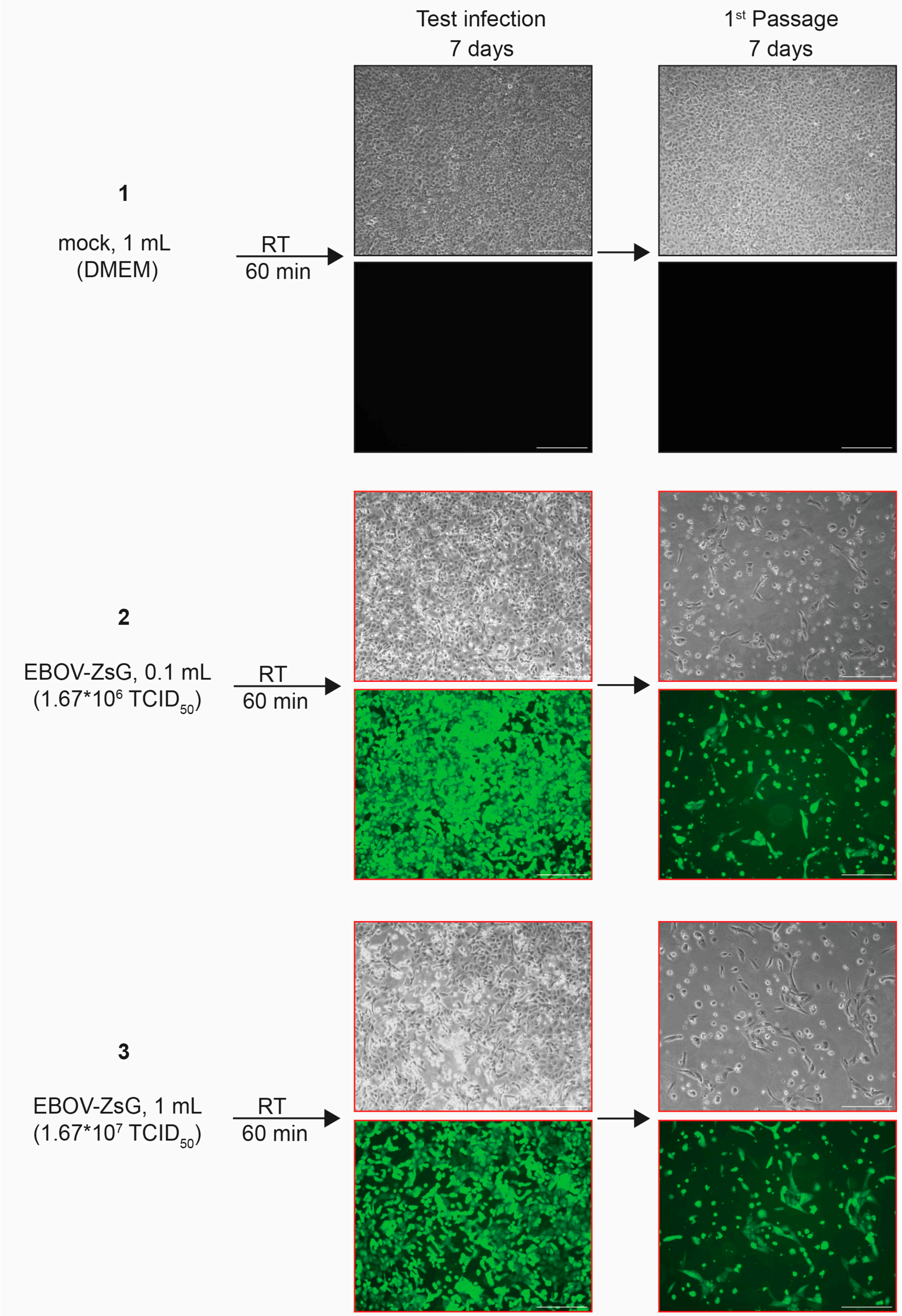

Figure S9. (1/6) Higher resolution versions of images from Figure 3. Scale bars = 250 μm.

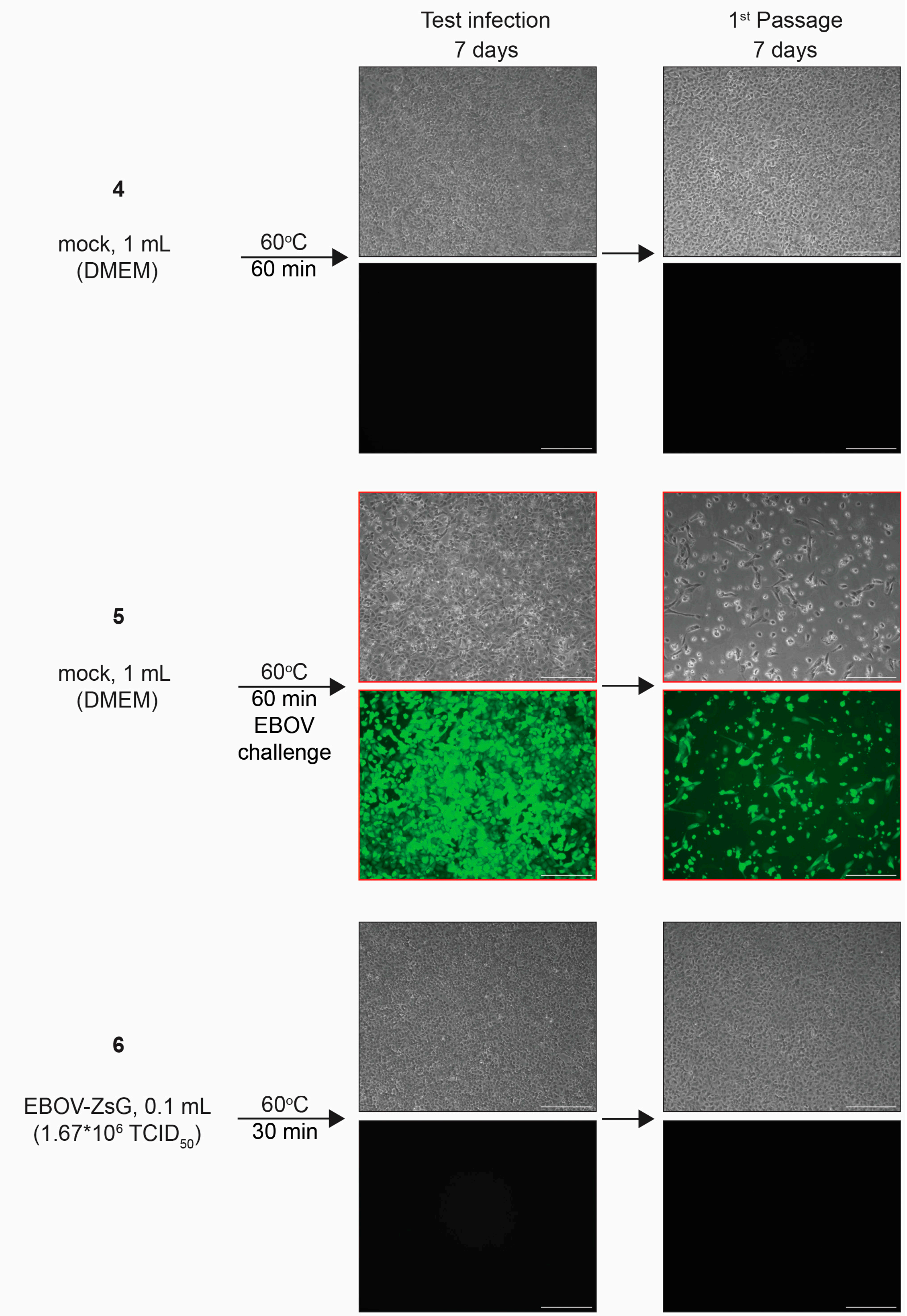

Figure S9. (2/6) Higher resolution versions of images from Figure 3. Scale bars = 250 μm.

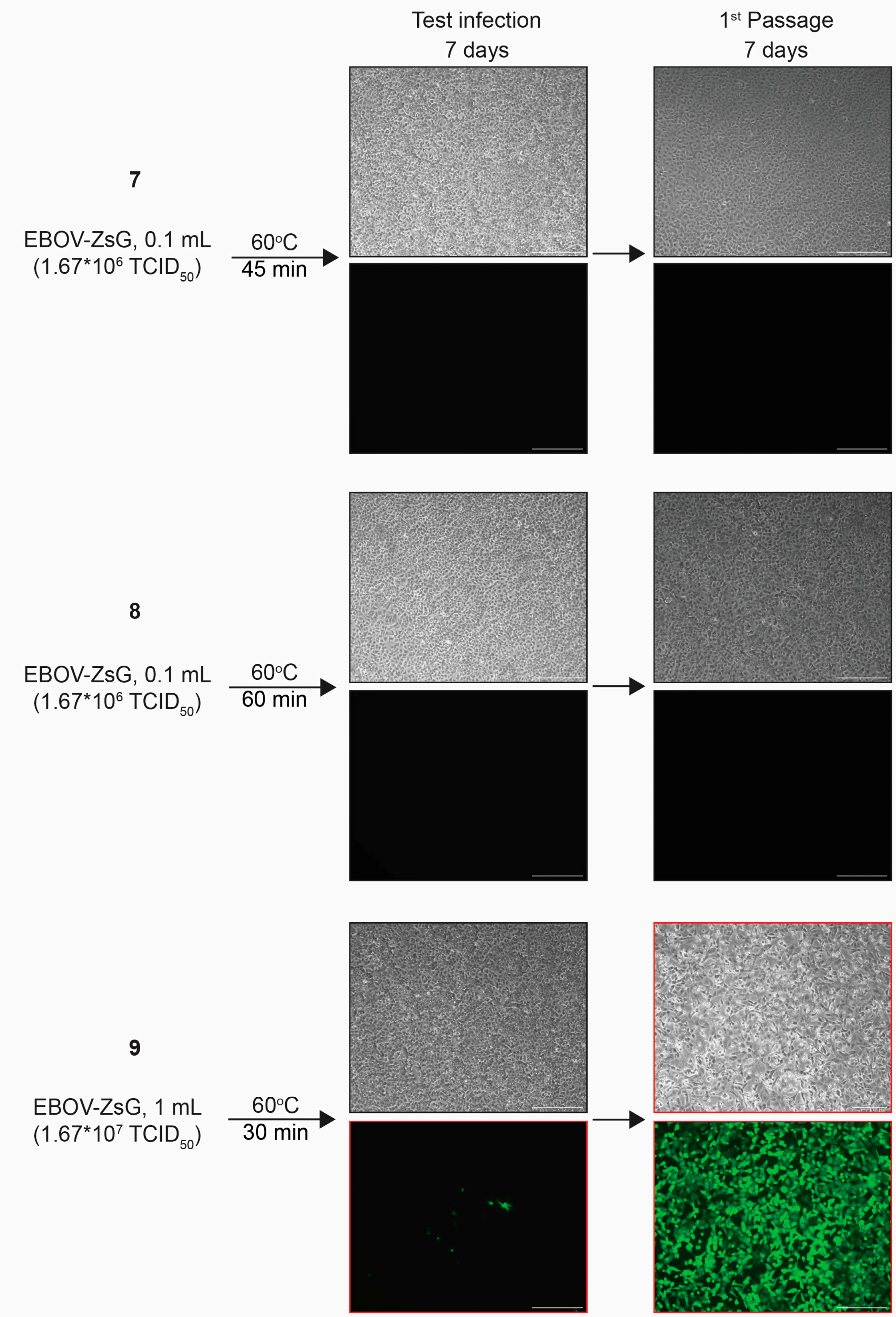

Figure S9. (3/6) Higher resolution versions of images from Figure 3. Scale bars = 250 μm.

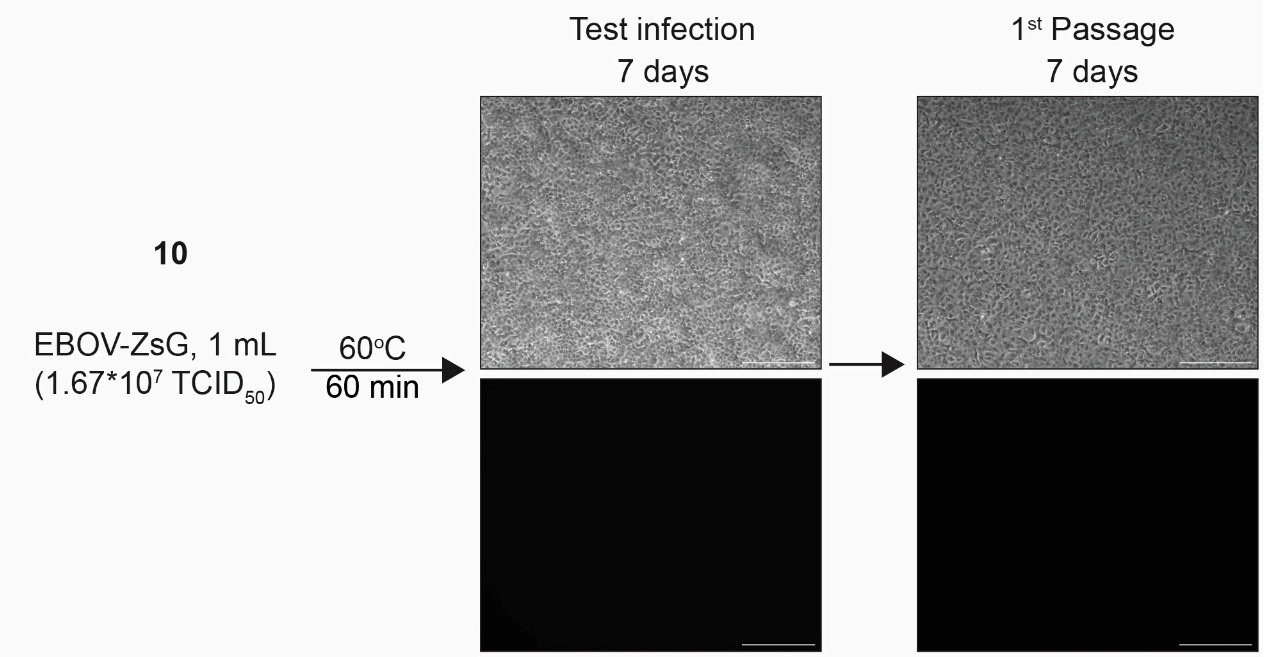

Figure S9. (4/6) Higher resolution versions of images from Figure 3. Scale bars = 250 μm.

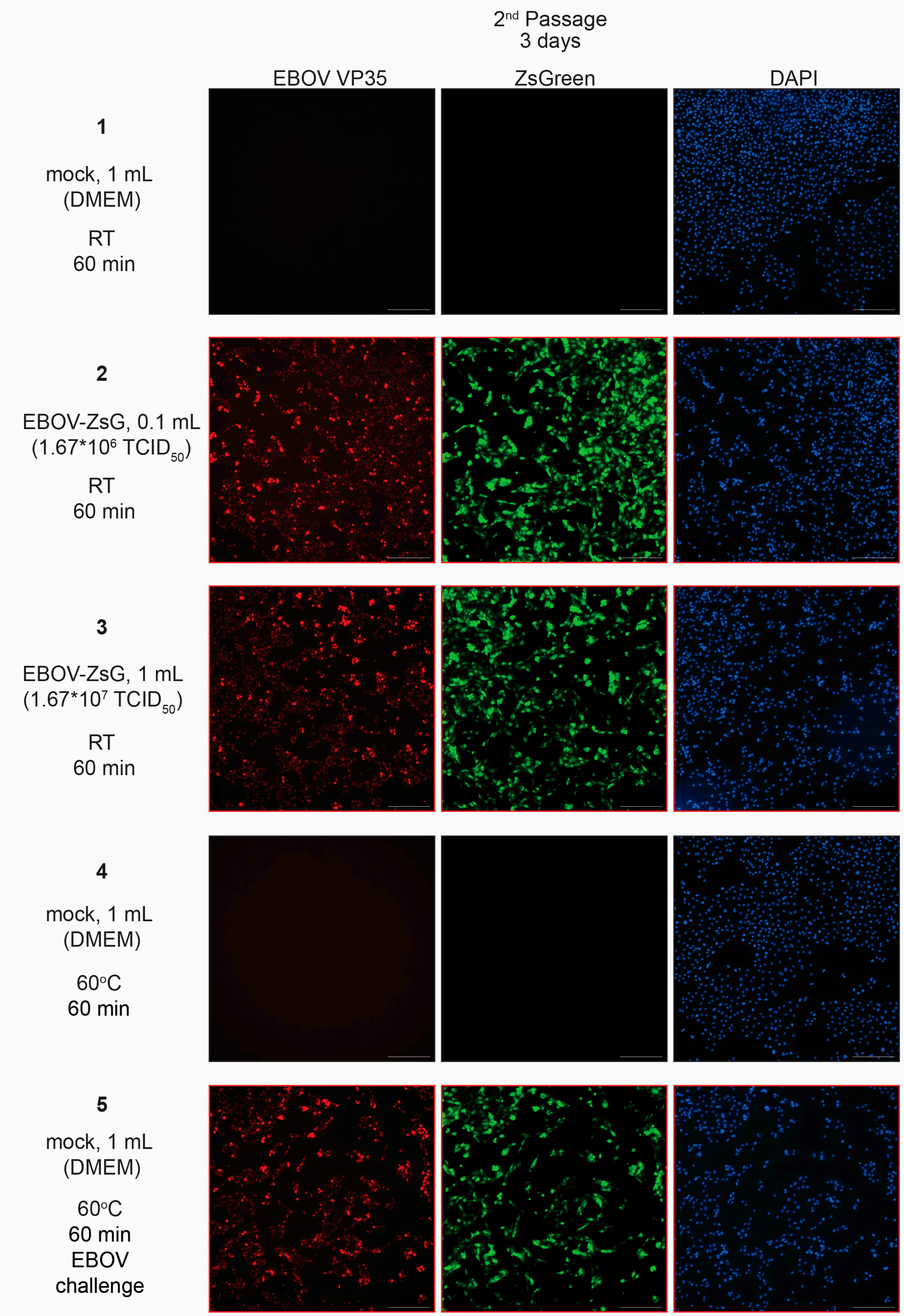

Figure S9. (5/6) Higher resolution versions of images from Figure 3. Scale bars = 250 μm.

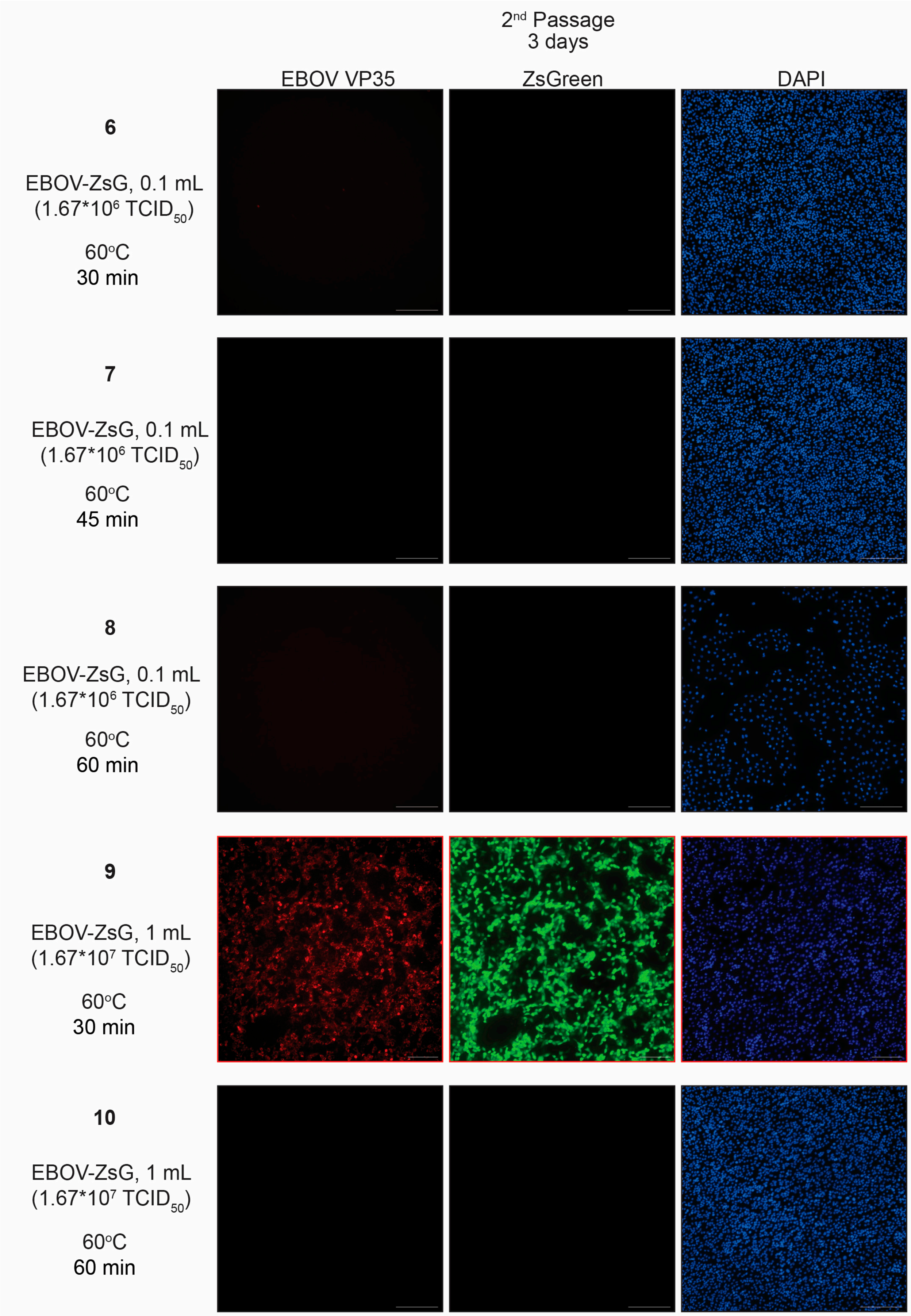

Figure S9. (6/6) Higher resolution versions of images from Figure 3. Scale bars = 250 μm.
